# P16^*INK4*a^ Deletion Ameliorates Damage of Intestinal Epithelial Barrier and Microbial Dysbiosis in a Stress-Induced Premature Senescence Model of *Bmi-1* Deficiency

**DOI:** 10.3389/fcell.2021.671564

**Published:** 2021-10-07

**Authors:** Jiawen Zhou, Chenxing Hou, Haiyun Chen, Ziyue Qin, Zi’an Miao, Jingyu Zhao, Qiuyi Wang, Min Cui, Chunfeng Xie, Rong Wang, Qing Li, Guoping Zuo, Dengshun Miao, Jianliang Jin

**Affiliations:** ^1^Research Center for Bone and Stem Cells, Department of Human Anatomy, Key Laboratory for Aging and Disease, The State Key Laboratory of Reproductive Medicine, Nanjing Medical University, Nanjing, China; ^2^Anti-Aging Research Laboratory, Friendship Plastic Surgery Hospital, Nanjing Medical University, Nanjing, China; ^3^Department of Nutrition and Food Safety, School of Public Health, Nanjing Medical University, Nanjing, China; ^4^Department of Science and Technology, Jiangsu Jiankang Vocational College, Nanjing, China

**Keywords:** inflammaging, TNF-α, *Desulfovibrio*, occludin, p16^*INK4a*^, Bmi-1

## Abstract

This study aimed to determine whether *Bmi-1* deficiency leads to intestinal epithelial barrier destruction and microbiota dysfunction, which members of the microbial community alter barrier function with age, and whether *p16*^*INK4a*^ deletion could reverse the damage of intestinal epithelial barrier and microbial dysbiosis. Intestines from *Bmi-1*–deficient (*Bmi-1^–/–^*), *Bmi-1* and *p16*^*INK4a*^ double-knockout (*Bmi-1^–/–^p16^*INK4a*–/–^*), and wild-type mice were observed for aging and inflammation. Duolink Proximity Ligation Assay, immunoprecipitation, and construction of *p16*^*INK4a*^ overexpressed adenovirus and the overexpressed plasmids of full-length, mutant, or truncated fragments for occludin were used for analyzing the interaction between p16^*INK4a*^ and occludin. High-throughput sequencing of V4 region amplicon of 16S ribosomal RNA was conducted using intestinal microbiota. We found *Bmi-1* deficiency destructed barrier structure, barrier function, and tight junction (TJ) in intestinal epithelium; decreased the TJ proteins; increased tumor necrosis factor α (TNF-α)–dependent barrier permeability; and up-regulated proinflammatory level of macrophages induced by intestinal microbial dysbiosis. The transplantation of fecal microbiota from wild-type mice ameliorated TJ in intestinal epithelium of *Bmi-1^–/–^* and *Bmi-1^–/–^p16^*INK4a*–/–^* mice. Harmful bacteria including *Desulfovibrio*, *Helicobacter*, and *Oscillibacter* were at a higher level in *Bmi-1^–/–^* mice. More harmful bacteria *Desulfovibrio* entered the epithelium and promoted macrophages-secreted TNF-α and caused TNF-α–dependent barrier permeability and aging. Accumulated p16^*INK4a*^ combined with occludin at the 1st–160th residue in cytoplasm of intestinal epithelium cells from *Bmi-1^–/–^* mice, which blocked formation of TJ and the repair of intestinal epithelium barrier. *P16*^*INK4a*^ deletion could maintain barrier function and microbiota balance in *Bmi-1^–/–^* mice through strengthening formation of TJ and decreasing macrophages-secreted TNF-α induced by *Desulfovibrio* entering the intestinal epithelium. Thus, Bmi-1 maintained intestinal TJ, epithelial barrier function, and microbiota balance through preventing senescence characterized by p16^*INK4a*^ accumulation. The clearance of p16^*INK4a*^-positive cells in aging intestinal epithelium would be a new method for maintaining barrier function and microbiota balance. The residues 1–160 of occludin could be a novel therapeutic target for identifying small molecular antagonistic peptides to prevent the combination of p16^*INK4a*^ with occludin for protecting TJ.

## Introduction

Mammalian intestine is the critical site of digestion, absorption and assimilation, and a highly immune-active ecosystem that harbors and preserves a large abundance of commensal microorganisms. Intestinal epithelium is the first defensive barrier against environmental and microbial attacks by maintaining a tight physical barrier and executing several critical innate immune functions (Patankar and Becker, 2020).

Aging is an intrinsic physiological process characterized by a gradual function decline in the organs, including intestine and its microbiota (Lin et al., 2019). With aging-dependent decrease in the intestinal barrier function, microbial productions including proinflammatory factors enter the bloodstream, triggering systemic inflammation (Thevaranjan et al., 2017). Besides, aging-related microbial imbalance increases intestinal permeability. Age-related microbial imbalance and intestinal barrier dysfunction have been associated with various senescence-associated intestinal and systemic diseases including inflammatory bowel diseases, celiac disease, type 1 diabetes, obesity, and Alzheimer disease (Lee, 2015). Previous study shows that tumor necrosis factor α (TNF-α) is the main proinflammatory cytokine that causes the destruction of epithelial tight junction (TJ), increases intestinal epithelial permeability, and aggravates senescence-associated systemic inflammation; however, which members of the microbial community alter barrier function with age have not been yet identified (Thevaranjan et al., 2017). The tumor suppressor protein p16^*INK4a*^ (hereafter referred to as p16), which is encoded by *INK4a* locus, is often transcriptionally activated in senescent cells and seen as a classical aging marker. P16 is up-regulated in multiple tissues during aging and contributes to senescence-associated decline in tissue function and regenerative capacity (Helman et al., 2016). However, whether accumulated p16 plays a critical role in damaging intestinal epithelial barrier and microbial homeostasis in inflammaging process is unclear.

B-cell–specific Moloney murine leukemia virus insertion region 1 (Bmi-1) is associated with senescence and cell cycle regulation. It regulates cell cycle and delays cell aging by inhibiting *INK4a/ARF* gene locus (Chen et al., 2020). Previous study on pig suggests that Bmi-1 is one of the markers of fast-cycling and quiescent intestinal stem cells (ISCs) that drives self-renewal of intestinal epithelial cells, increasing intestinal epithelial cell proliferation by stimulating WNT/β-catenin signaling (Li et al., 2018). Bmi-1–positive ISCs are critical for intestinal epithelium to maintain its function as a primary barrier (Smith et al., 2018). Loss of *Bmi-1* in mice reduces proliferation in the ISC compartment accompanied by p16 accumulation (Lopez-Arribillaga et al., 2015). Several lines of evidence demonstrate that *Bmi-1–*deficient mouse is a stress-induced premature senescence (SIPS) model that appears frail with malnutrition and shortened life span (Liu et al., 2009; Zhang et al., 2010; Jin et al., 2014, 2017; Xie et al., 2015; Chen et al., 2020). However, whether *Bmi-1* deficiency could cause damage of intestinal epithelial barrier and microbial dysbiosis is unclear. It is also unknown if *p16* deletion could ameliorate the damage of intestinal epithelial barrier and microbial dysbiosis in *Bmi-1^–/–^* mice.

Herein we report that Bmi-1 maintained intestinal TJ, epithelial barrier function, and microbiota balance through preventing senescence characterized by p16 accumulation. Accumulated p16 combined with occludin at the 1st to 160th residue in the cytoplasm of aging intestinal epithelium cells, which blocked the repair of intestinal epithelium barrier. *P16* deletion could maintain barrier function and microbiota balance in *Bmi-1^–/–^* mice through strengthening formation of TJ and decreasing macrophages-secreted TNF-α induced by *Desulfovibrio* entering the intestinal epithelium.

## Results

### Damage of Barrier Structure and Dysfunction in Intestinal Epithelium Ameliorated by *p16* Deletion in *Bmi-1^–/–^* Mice

**FIGURE 1:**
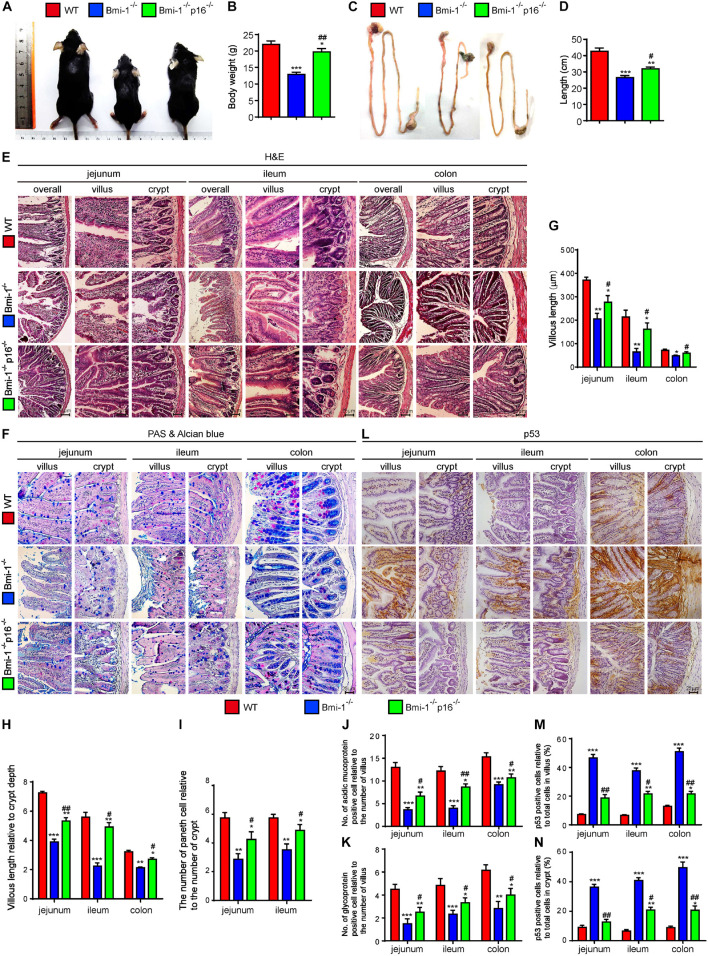
*P16* deletion improved damage of barrier structure and dysfunction in intestinal epithelium in *Bmi-1^–/–^* mice. The experiments were carried out on the 7-week-old *Bmi-1^–/–^*, *Bmi-1^–/–^p16^–/–^*, and WT mice. **(A)** Representative appearances. **(B)** Body weight (g). **(C)** Whole view of the intestine. **(D)** Intestinal length (cm). **(E,F)** H&E and AB-PAS staining of jejunum, ileum, and colon. **(G)** Villous length (μm). **(H)** Villous length relative to crypt depth. **(I)** The number of Paneth cells relative to the number of crypts. **(J)** The number of acidic mucoprotein positive cells. **(K)** Glycoprotein-positive cells relative to the number of villus. **(L)** Representative micrographs of paraffin-embedded intestinal sections immunohistochemical staining for p53, with hematoxylin staining the nucleus. **(M,N)** Percentage of p53-positive cells relative to the total cells. Six mice per group were used for experiments. Statistical analysis was performed with one-way ANOVA test. Values are mean ± SEM from six determinations per group, *p < 0.05, **p < 0.01, ***p < 0.001 compared with the WT group; ^#^*p* < 0.05, ^##^*p* < 0.01 compared with the *Bmi-1*^–/–^ group.

Bmi-1 and p16 were widely expressed in epithelial cells of jejunum, ileum, and colon (Supplementary Figures 1, 2). To examine if *p16* deletion ameliorated the damage of barrier structure and dysfunction in intestinal epithelium in *Bmi-1^–/–^* mice, body size and weight, and intestinal length, histological structure and secretory function were observed in 7-week-old *Bmi-1^–/–^* mice, *Bmi-1^–/–^p16^–/–^* mice, and wild-type (WT) mice. Results showed that besides the smaller body size and weight, *Bmi-1^–/–^* mice also showed shorter intestinal length (especially the ileum) in comparison with WT mice. Deletion of *p16* significantly rescued the body size, weight, and intestinal length caused by *Bmi-1* deficiency (Figures 1A–D). Significant decreases were observed in the villus length, the ratio of villus length to crypt, the number of Paneth cells, and the number of acid mucin and glycoprotein in *Bmi-1^–/–^* mice compared with WT mice. *P16* deletion significantly rescued the abnormalities in the villus length, the ratio of villus length to crypt, the number of Paneth cells, and the number of acid mucin and glycoprotein observed in *Bmi-1^–/–^* mice (Figures 1E–K). Ki67-positive cells significantly decreased after *Bmi-1* deletion, which was rescued by *p16* knockout (Supplementary Figure 3). P53 significantly increased in jejunum, ileum, and colon of *Bmi-1^–/–^* mice and then down-regulated by *p16* deletion (Figures 1L–N). These results demonstrated that *p16* deletion ameliorated the damage of barrier structure and dysfunction in intestinal epithelium caused by *Bmi-1* deficiency.

### Destruction of Tight Junction and Increase in Barrier Permeability in Intestinal Epithelium Ameliorated by *p16* Deletion in *Bmi-1^–/–^* Mice

**FIGURE 2:**
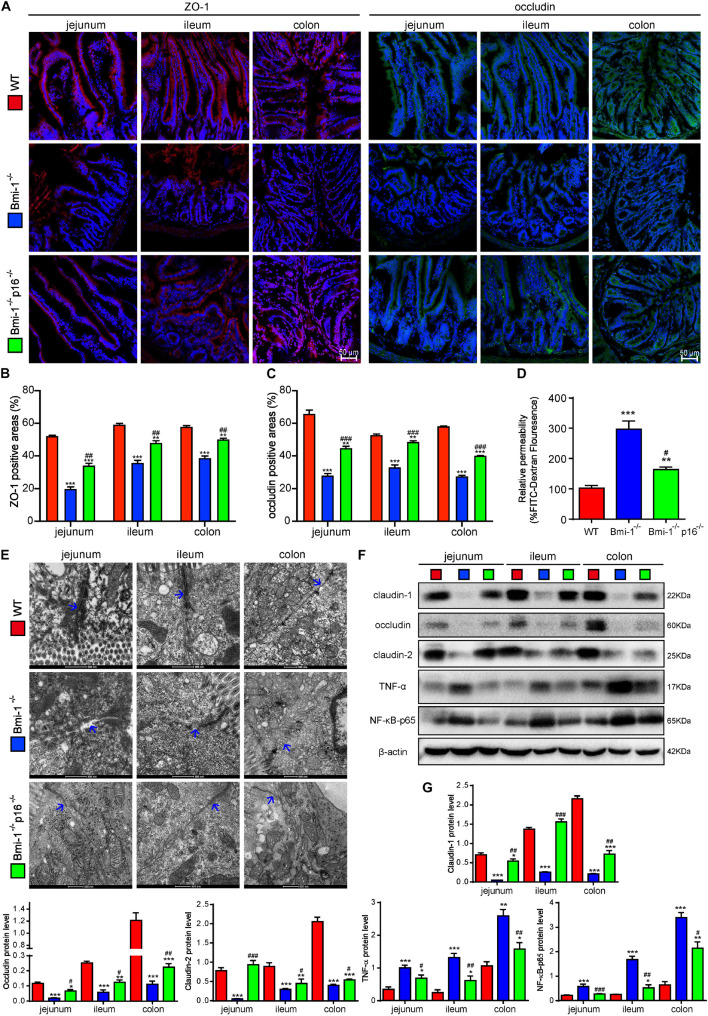
*P16* deletion improved destruction of TJ and increasing barrier permeability in intestinal epithelium in *Bmi-1^–/–^* mice. The experiments were carried out on the 7-week-old *Bmi-1^–/–^*, *Bmi-1^–/–^p16^–/–^*, and WT mice. **(A)** Representative micrographs showing immunofluorescence for ZO-1 and occludin, with DAPI staining the nuclei. **(B)** Percentage of ZO-1–positive areas relative to the total area. **(C)** Percentage of occludin-positive areas relative to the total area. **(D)** Intestinal FITC–dextran transmittance showing intestinal permeability. **(E)** Representative micrographs showing transmission electron microscope for the epithelial cells of jejunum, ileum, and colon, with blue arrow showing the bottom of the epithelial TJ. **(F)** Western blots for claudin-1, occludin, claudin-2, TNF-α, and NF-κB–p65 in the epithelial cells of jejunum, ileum, and colon; β-actin was used as the loading control. **(G)** Protein levels relative to β-actin were assessed by densitometric analysis. Six mice per group were used for experiments. Statistical analysis was performed with one-way ANOVA test. Values are mean ± SEM from six determinations per group, *p < 0.05, **p < 0.01, ***p < 0.001 compared with the WT group; ^#^*p* < 0.05, ^##^*p* < 0.01, ^###^*p* < 0.001compared with the *Bmi-1*^–/–^ group.

To investigate if *p16* deletion ameliorated the destruction of TJ and the increase of barrier permeability in *Bmi-1*^–/–^ mice, the key TJ protein ZO-1, tight junctional structure, and several primary TJ proteins including claudin-1, occludin, and claudin-2 in the epithelia of jejunum, ileum, and colon were detected and analyzed. Moreover, peripheral blood was collected and detected fluorescein isothiocyanate (FITC) fluorescence for evaluating intestinal permeability at the fourth hour after oral gavage of FITC–dextran. In *Bmi-1^–/–^* mice compared with WT mice, significant decreases were observed in ZO-1– and occludin-positive areas (Figures 2A–C and Supplementary Figures 4A,B) and protein levels of claudin-1, occludin, and claudin-2 in epithelia (Figures 2F,G); meanwhile, a significant increase was observed in FITC fluorescence for evaluating intestinal permeability (Figure 2D). It was also observed in electron micrographs that the bottom of the epithelial TJ was cracked in epithelia of jejunum, ileum, and colon (Figure 2E). *P16* deletion significantly increased the expressions of TJ proteins, maintained the tight junctional structure, and decreased the intestinal permeability (Figure 2). These results demonstrated that *p16* deletion improved the destruction of TJ and decreased barrier permeability in intestinal epithelium caused by *Bmi-1* deficiency.

### Accumulated *p16* in *Bmi-1^–/–^* Mice Combined With Occludin in the Cytoplasm of Intestinal Epithelium Cells for Blocking the Repair of Tight Junction

**FIGURE 3:**
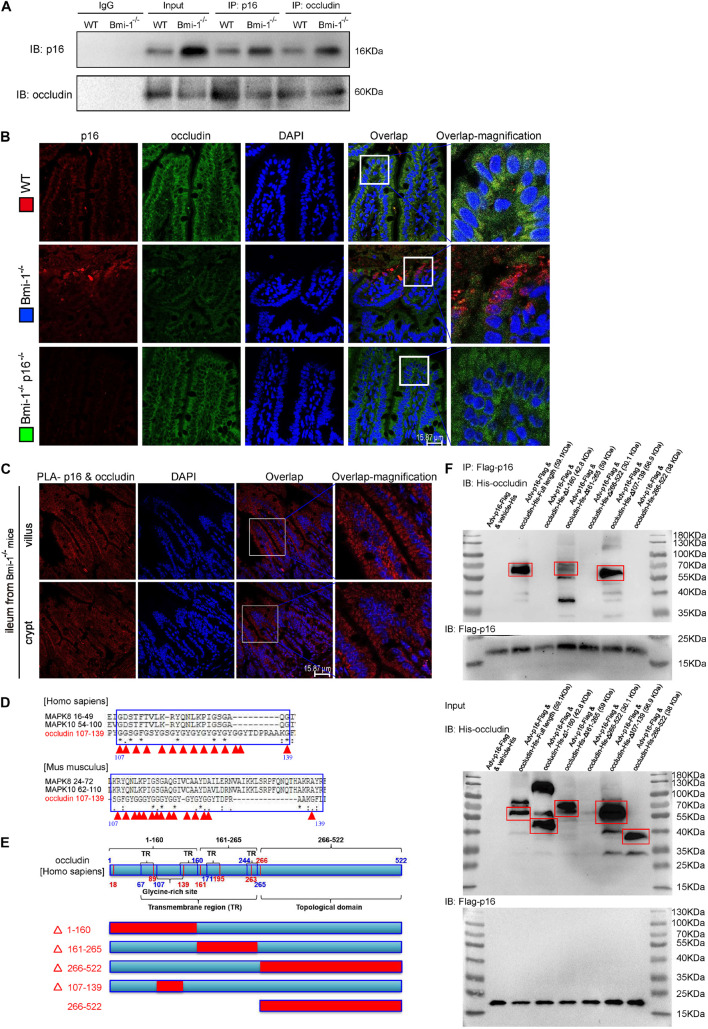
*P16* combined with occludin in the cytoplasm of intestinal epithelium cells in *Bmi-1^–/–^* mice for blocking reparation of TJ. The experiments were carried out on the 7-week-old *Bmi-1^–/–^*, and WT mice. **(A)** Intestinal epithelial proteins from WT and *Bmi-1^–/–^* mice were extracted for anti-p16 or anti-occludin immunoprecipitation. Western blots were used for detecting p16 and occludin. **(B)** Representative micrographs showing immunofluorescence double-labeling of p16 and occludin in ileum from 7-week-old *Bmi-1^–/–^, Bmi-1^–/–^p16^–/–^* and WT mice. **(C)** Representative micrographs of Duolink PLA for interaction between p16 and occludin in villus and crypt of ileum from *Bmi-1^–/–^* mice, with DAPI staining nuclei. **(D)** Analysis with https://www.uniprot.org/align showed an extremely similar domain among occludin, MAPK8, and MAPK10 in human and mouse, with red triangle showing glycine site, “*” indicating a single and fully conserved residue, “:” indicating residue with very similar properties, and “.” indicating residue that is weakly similar. **(E)** Protein domain of human occludin. **(F)** The 239T cells were transfected with *Flag-p16* (human) overexpressed adenovirus and the His-tagged plasmid of full length, 1–160–mutant (△1–160), 161–265–mutant (△161–265), 266–522–mutant (△266–522), 107–139–mutant (△107–139), or 266–522–truncated fragment for human *occludin*. Cell proteins were extracted for anti–Flag-Tag immunoprecipitation and detected anti-Flag and anti-His antibodies with Western blots. The input proteins also detected anti-Flag and anti-His antibodies with Western blots.

To investigate how *p16* deletion ameliorated intestinal epithelium barrier function in *Bmi-1*^–/–^ mice, we conducted immunoprecipitation of p16 and occludin, and validated p16 could combine with occludin (Figure 3A). P16 expression in *Bmi-1^–/–^* ileum was higher than WT ileum; however, occludin expression in *Bmi-1^–/–^* ileum was lower than WT ileum in input and Immunoprecipitation (IP) samples (Figure 3A). The results of immunofluorescence colocalization and Duolink Proximity Ligation Assay showed p16 bound to occludin in the cytoplasm of intestinal epithelium cells in *Bmi-1*^–/–^ mice (Figures 3B,C). Our results showed that p16 expressed in the nucleus and cytoplasm, especially more concentrated in the cytoplasm, and interacted with occludin in the cytoplasm (Figures 3B,C). Compared with the *Bmi-1^–/–^* mice, p16 expression was obviously decreased in epithelium cells of *Bmi-1^–/–^p16^–/–^* and WT mice (Figure 3B and Supplementary Figure 2). It has been reported that N-terminal of *p16* bound to the N-terminal region of JNK1 (also known as MAPK8) or the N-terminal region of JNK3 (also known as MAPK10), which contain the glycine-rich site (Choi et al., 2005). Analysis with https://www.uniprot.org/align showed an extremely similar domain among human occludin (residues 107–139), MAPK8 (residues 16–49), and MAPK10 (residues 54–100) and also showed an extremely similar domain among mouse occludin (residues 107–139), MAPK8 (residues 24–72), and MAPK10 (residues 62–110). Besides, the 107th–139th residues are also a glycine-rich site of occludin (Figure 3D and Supplementary Information 2, 3).

To determine which domain of occludin combined with p16, 293T cells were transfected with green fluorescent protein (GFP)-labeled *Flag-p16* overexpressed adenovirus and then with vehicle–His-tagged vector (negative control), His-tagged overexpressed plasmids of full-length, mutant, or truncated fragments for occludin, respectively. Cell extracts were immunoprecipitated with anti-Flag antibody, and the precipitated proteins were detected by immunoblotting with anti-His or anti-Flag antibody. As was shown from the results of immunoprecipitation, p16 combined with occludin at the residues 1–160 rather than limited to residues 107–139. Because the 266th–522nd mutant plasmid did not express protein, we constructed the 266th–522nd truncated fragment and found that p16 did not combine with this domain (Figures 3E,F). Thus, these results suggested that accumulated p16 combined with occludin at the residues 1–160 in the cytoplasm of aging intestinal epithelium cells in *Bmi-1*^–/–^ mice for blocking the repair of intestinal epithelium barrier.

### Accumulated p16 in Cacao-2 Cells Blocked the Repair of Tight Junction After Tight Junction Was Damaged by TNF-α

**FIGURE 4:**
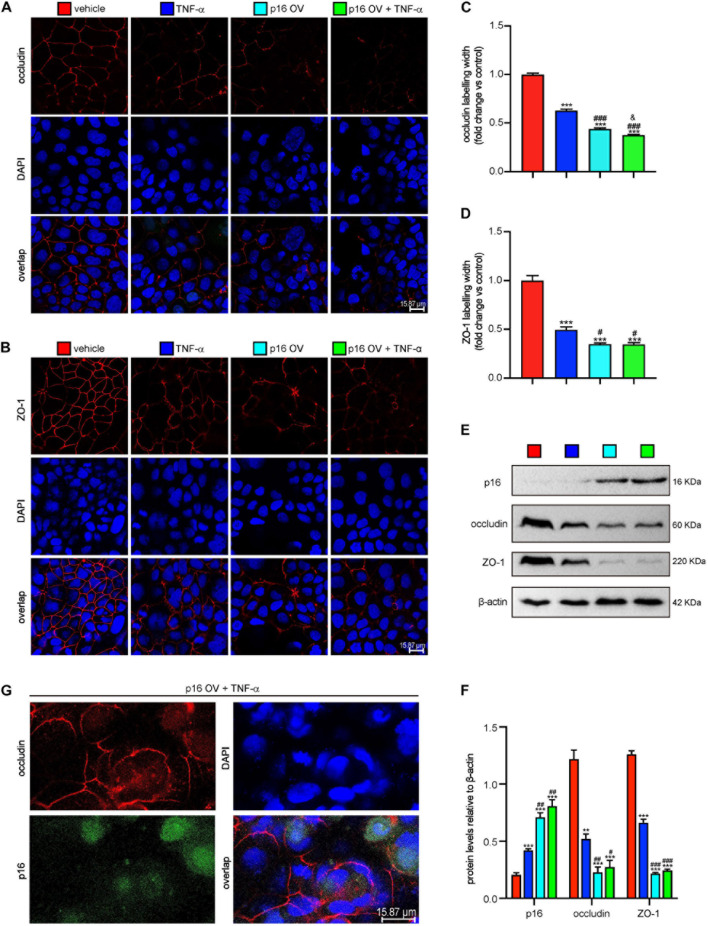
Accumulated p16 in Cacao-2 cells blocked the repair of TJ after TJ was damaged by TNF-α. The Cacao-2 cells were transfected with GFP-labeled *Flag-p16–*overexpressed (*p16*-OV) or vehicle adenovirus and then treated with or without 10 ng/mL TNF-α for 24 h. Immunofluorescence staining was introduced to detect the expressions of occludin and ZO-1. **(A)** Representative micrographs showing immunofluorescence for occludin, with DAPI staining the nuclei. **(B)** Representative micrographs showing immunofluorescence for ZO-1, with DAPI staining the nuclei. **(C)** Occludin positive width relative to control group. **(D)** ZO-1 positive width relative to control group. **(E)** Western blots for p16, occludin, and ZO-1 in Cacao-2 cells, and β-actin was used as the loading control. **(F)** Protein levels relative to β-actin were assessed by densitometric analysis. Cell experiments were performed with three biological repetitions per group. Statistical analysis was performed with one-way ANOVA test. Values are mean ± SEM from six determinations per group, **p < 0.01, ***p < 0.001 compared with the vehicle group; ^#^*p* < 0.05, ^##^*p* < 0.01, ^###^*p* < 0.001 compared with TNF-α–treated group, ^&^*P* < 0.05 compared with the *p16*-OV group. **(G)** Representative micrographs showing occludin and GFP-labeled p16 in *p16*-OV and TNF-α–treated cells, with DAPI staining the nuclei.

To further determine if the p16–occludin interaction blocks the repair of TJ, Cacao-2 cells were transfected with GFP-labeled *Flag-p16* overexpressed adenovirus and then were given TNF-α stimulation to damage the TJ. Immunofluorescence staining and Western blot were used to detect the expression levels of occludin and ZO-1. Immunofluorescence staining was also used to observe the interaction of p16 and occludin. Our results showed that in comparison with vehicle treatment, TNF-α treatment or *p16* overexpression damaged TJ and decreased the expressions of occludin and ZO-1 (Figures 4A–F). Compared with vehicle–adenovirus and TNF-α treatment, *p16* overexpressed adenovirus, and TNF-α treatment further damaged TJ and decreased the expressions of occludin and ZO-1 (Figures 4A–F). We also observed that when the TJ between cells was destroyed, more occludin expression was found in cytoplasm and colocalized with p16 (Figure 4G).

### TNF-α–Dependent Epithelial Barrier Destruction Ameliorated by *p16* Deletion in *Bmi-1^–/–^* Mice

**FIGURE 5:**
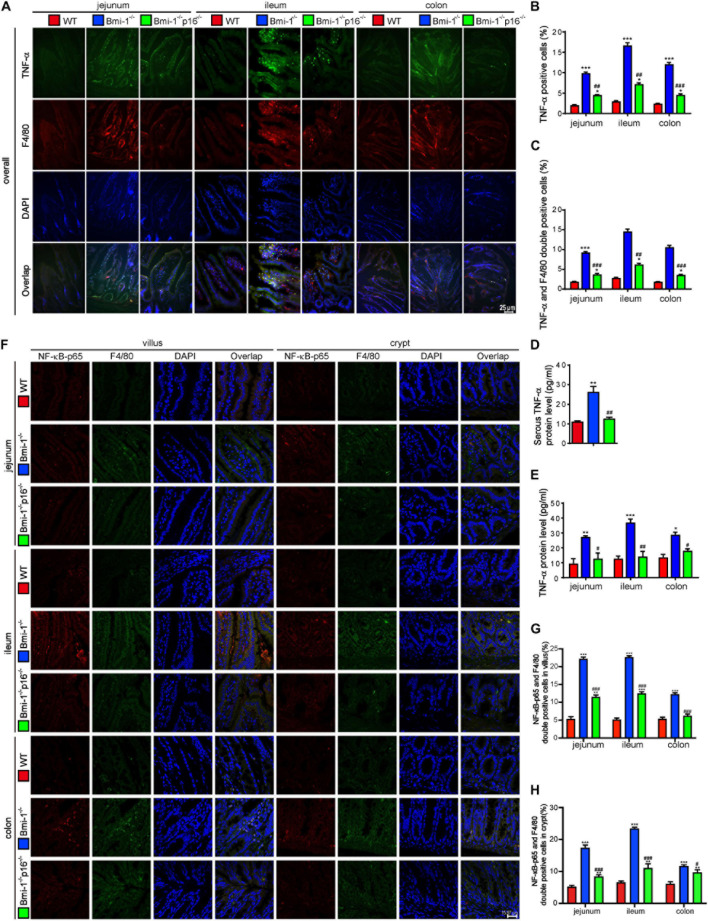
*P16* deletion improved TNF-α–dependent epithelial barrier destruction in *Bmi-1^–/–^* mice. The experiments were carried out on the 7-week-old *Bmi-1^–/–^*, *Bmi-1^–/–^p16^–/–^*, and WT mice. **(A)** Representative micrographs showing immunofluorescence for TNF-α and F4/80, with DAPI staining the nuclei. **(B)** Percentage of TNF-α–positive cells. **(C)** Percentage of TNF-α and F4/80 double-positive cells. **(D)** Serous TNF-α protein level (pg/mL) was detected with ELISA assay. **(E)** TNF-α protein levels in jejunum, ileum, and colon (pg/mL) were detected with ELISA assay. **(F)** Representative micrographs showing immunofluorescence for NF-κB–p65 and F4/80, with DAPI staining the nuclei. **(G,H)** Percentage of NF-κB–p65 and F4/80 double-positive cells or areas in villus and crypt. Six mice per group were used for experiments. Statistical analysis was performed with one-way ANOVA test. Values are mean ± SEM from six determinations per group, *p < 0.05, **p < 0.01, ***p < 0.001 compared with the WT group; ^#^*p* < 0.05, ^##^*p* < 0.01, ^###^*p* < 0.001 compared with the *Bmi-1*^–/–^ group.

To determine if *p16* deletion ameliorated the TNF-α–dependent epithelial barrier destruction in *Bmi-1*^–/–^ mice, TNF-α–positive cells or areas; TNF-α and F4/80 double-positive macrophages; TNF-α protein levels in jejunum, ileum, and colon; and serous TNF-α protein levels of mice were detected and analyzed. Results showed that TNF-α–positive cells; TNF-α and F4/80 double-positive macrophages; TNF-α protein levels in jejunum, ileum, and colon; and serous TNF-α protein levels were significantly increased in *Bmi-1^–/–^* mice compared with WT mice; however, they were significantly reduced in *Bmi-1^–/–^p16^–/–^* mice compared with the *Bmi-1^–/–^* mice (Figures 5A–E). Then, nuclear factor κB (NF-κB)–p65–positive cells and NF-κB–p65 and F4/80 double-positive macrophages were detected in jejunum, ileum, and colon, and results showed that they were significantly increased in *Bmi-1^–/–^* mice compared with WT mice, while significantly reduced in *Bmi-1^–/–^p16^–/–^* mice compared with the *Bmi-1^–/–^* mice (Figures 5F–H). It suggested that the proinflammatory level of macrophages in intestines was up-regulated in *Bmi-1^–/–^* mice compared with WT mice and ameliorated by *p16* deletion.

### Up-Regulated Proinflammatory Level of Macrophages Induced by Change of Intestinal Microbiota Ameliorated by *p16* Deletion in *Bmi-1^–/–^* Mice

**FIGURE 6:**
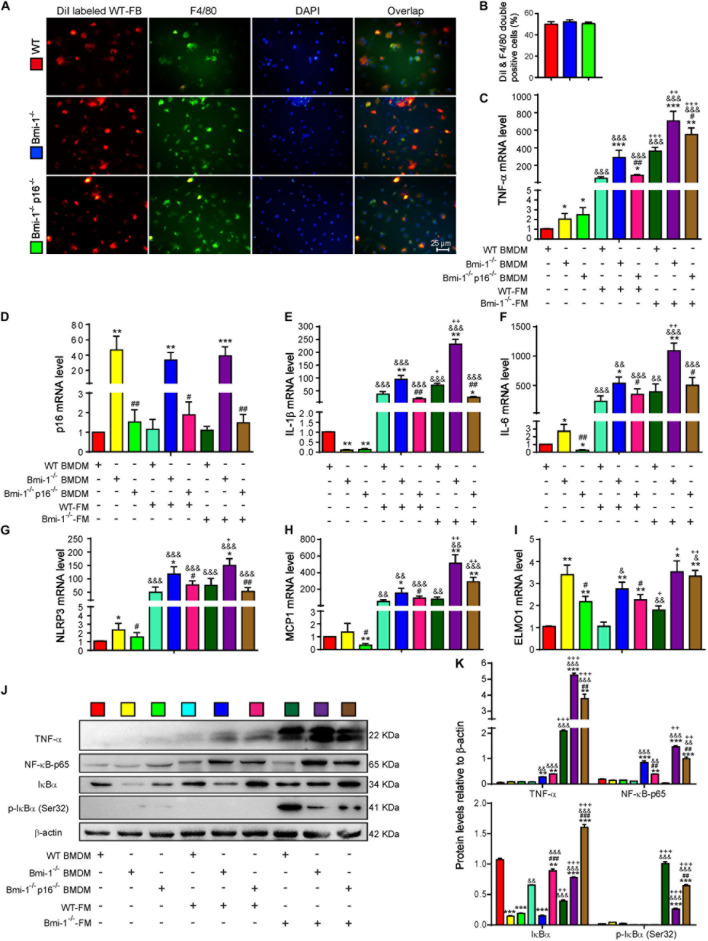
*P16* deletion improved up-regulated proinflammatory level of macrophages induced by change of intestinal microbiota ameliorated in *Bmi-1^–/–^* mice. Fecal mixed bacteria (FB) were prepared using the fecal of 7-week-old WT and *Bmi-1*^–/–^ mice to infect BMDMs from WT, *Bmi-1*^–/–^, and *Bmi-1*^–/–^*p16*^–/–^ mice. **(A)** Representative micrographs showing immunofluorescence for F4/80 after Dil-labeled WT-FM–infected BMDMs, with DAPI staining the nuclei, red dots showing Dil-labeled WT-FB, and green dots showing F4/80. **(B)** Percentage of DiI and F4/80 double-positive cells. **(C–I)** Tumor necrosis factor (*TNF-*α), *p16*, interleukin (*IL-1*β), *IL-6*, NACHT, LRR, and PYD domain–containing protein 3 (*NLRP3*) and monocyte chemoattractant protein 1 (*MCP1*) and engulfment and cell motility protein 1 (*ELMO1*) mRNA levels in BMDMs by real-time RT-PCR, calculated as the ratio to *β-actin* mRNA and expressed relative to WT. **(J)** Western blots of BMDM extracts showing TNF-α, NF-κB–p65, IκBα, and p-IκBα (Ser32); β-actin was used as the loading control. **(K)** Protein levels relative to β-actin were assessed by densitometric analysis. Cell experiments were performed with three biological repetitions per group. Statistical analysis was performed with one-way ANOVA test. Values are mean ± SEM from six determinations per group, *p < 0.05, **p < 0.01, ***p < 0.001 compared with the WT group at the same treatment; ^#^*p* < 0.05, ^##^*p* < 0.01, ^###^*p* < 0.001 compared with the *Bmi-1*^–/–^ group at the same treatment; ^&^*p* < 0.05, ^&&^*p* < 0.01, ^&&&^*p* < 0.001 compared with the same genotyped BMDMs of vehicle-FM group; ^+^*p* < 0.05, ^++^*p* < 0.01, ^+++^*p* < 0.001 compared with the same genotyped BMDMs of the WT-FM group.

To investigate if *Bmi-1* deficiency and/or *p16* deletion changed the phagocytosis of macrophages, DiI (1,1’-dioctadecyl-3,3,3’,3’-tetramethylindocarbocyanine perchlorate)–labeled fecal mixed bacteria from WT mice (WT-FB) were used to infect bone marrow–derived macrophages (BMDMs) from *Bmi-1*^–/–^, *Bmi-1*^–/–^*p16*^–/–^, and WT mice. Immunofluorescence staining of macrophage marker F4/80 proved that there was no difference in phagocytosis of BMDMs from WT, *Bmi-1*^–/–^, and *Bmi-1*^–/–^*p16*^–/–^ mice (Figures 6A,B). This result suggested that Bmi-1 and/or p16 did not affect the phagocytosis of macrophages.

To further make clear if the up-regulated proinflammatory level of macrophages was associated with the change of intestinal microbiota in *Bmi-1^–/–^* mice, WT–fecal microbiota (FM), *Bmi-1^–/–^*–FM, and vehicle were administered to BMDMs from WT, *Bmi-1*^–/–^, and *Bmi-1*^–/–^*p16*^–/–^ mice. In comparison with the WT-FM–treated BMDMs of the same genotype, an increase was observed in the mRNA levels of *TNF-*α, interleukin (*IL*)*-1*β, *IL-6*, NACHT, LRR, and PYD domain–containing protein 3 (*NLRP3*), monocyte chemoattractant protein 1 (*MCP1*), and engulfment and cell motility protein1 (*ELMO1*) and in the protein levels of TNF-α, NF-κB–p65, IκBα, and p-IκBα (Ser32) in *Bmi-1^–/–^*–FM treated BMDMs, especially in *Bmi-1*–null BMDMs. Compared with vehicle of the same genotype, WT-FM and *Bmi-1^–/–^*–FM treatments significantly increased the above mRNA levels and protein levels, except the *ELMO1* mRNA level and IκBα and p-IκBα (Ser32) protein level (Figures 6C–K). These results suggested that the up-regulated proinflammatory level of macrophages was associated with the change of intestinal microbiota in *Bmi-1^–/–^* mice.

To determine if *p16* deletion ameliorated proinflammatory level of macrophages activated by aging-associated intestinal microbiota in *Bmi-1^–/–^* mice, WT-FM, *Bmi-1^–/–^*–FM, and vehicle were administered to BMDMs from *Bmi-1*^–/–^, *Bmi-1*^–/–^*p16*^–/–^, and WT mice. Results showed that whatever the treatment was with WT-FM or *Bmi-1^–/–^*–FM, an increase was observed in the mRNA levels of *TNF-*α, *IL-1*β, *IL-6*, *NLRP3*, *MCP1*, and *ELMO1* and in the protein levels of TNF-α and NF-κB–p65 in *Bmi-1^–/–^* BMDMs compared with WT BMDMs; however, they were significantly reduced in *Bmi-1^–/–^p16^–/–^* mice compared with the *Bmi-1^–/–^* mice (Figures 6C–K). These results suggested that the proinflammatory level of macrophages activated by intestinal microbiota was ameliorated by *p16* deletion in *Bmi-1^–/–^* mice.

### Fecal Microbiota Transplantation From Wild-Type Mice Ameliorates Tight Junction in Intestinal Epithelium of *Bmi-1*^–/–^ and *Bmi-1*^–/–^*p16*^–/–^ Mice

To determine if the damage of TJ in intestinal epithelium was associated with microbial dysbiosis, FM from 3- to 4-week-old WT mice were transplanted (WT-FMT) to WT, *Bmi-1*^–/–^, and *Bmi-1*^–/–^*p16*^–/–^ mice by gavage every other day and lasted for 21 days. Results showed that compared with the WT mice without or with FMT, ZO-1 and occludin expression in ileum epithelium were obviously decreased in *Bmi-1*^–/–^ mice without or with WT-FMT; however, they were significantly increased in *Bmi-1^–/–^p16^–/–^* mice without or with WT-FMT compared with the *Bmi-1^–/–^* mice without or with WT-FMT, respectively (Supplementary Figures 4A–D). Compared with the *Bmi-1*^–/–^ mice or *Bmi-1^–/–^p16^–/–^* mice without WT-FMT, ZO-1 and occludin expressions in ileum epithelium were obviously increased in *Bmi-1*^–/–^ mice or *Bmi-1^–/–^p16^–/–^* mice with WT-FMT, respectively (Supplementary Figures 4A–D). These results suggested that the destructive degree of TJ was regulated by intestinal microbiota.

### Intestinal Microbial Dysbiosis Ameliorated by *p16* Deletion in *Bmi-1^–/–^* Mice

**FIGURE 7:**
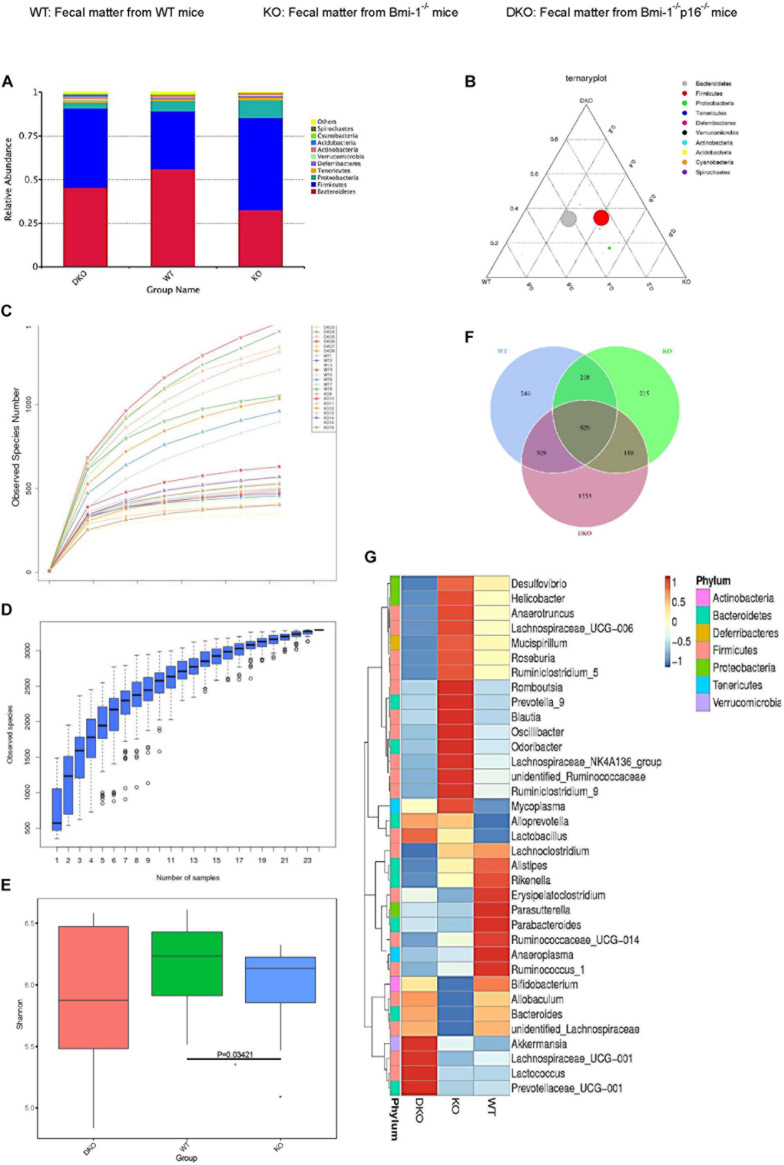
*P16* deletion improved intestinal microbial dysbiosis in *Bmi-1^–/–^* mice. High-throughput sequencing of V4 region amplicon of 16S rRNA from intestinal microbiota was conducted using the fecal of 7-week-old WT, *Bmi-1*^–/–^ (KO), and *Bmi-1*^–/–^*p16*^–/–^ (DKO) mice. **(A)** Principal components at the phylum level in the intestinal microbiota of WT, *Bmi-1*^–/–^, and *Bmi-1*^–/–^*p16*^–/–^ mice. Relative contribution of the top 10 phylum of each sample was shown; “others” represents the sum of abundance except the top 10 phylum, *n* = 8. **(B)** Proportion of intestinal microbiota in 7-week-old WT, KO, and DKO mice at the phylum level. Circle represents a taxonomy at the level of phylum; the size of the circle represents the abundance of the microbiota at the phylum level. **(C)** Abundance display curve of intestinal microbiota in WT, KO, and DKO mice. Abscissa represents sequencing depth; ordinate represents the number of species found. **(D)** Box plot showing abundance dilution. The end of the curve is close to gentle, indicating that the sample size of sequencing meets the requirements of diversity. **(E)** The observed species number and α-diversity index of intestinal bacteria were examined. Significant differences are indicated: Wilcoxon rank sum test, *n* = 8 per group, *p < 0.05, compared with the WT group. **(F)** Venn diagrams demonstrate the number of species shared among WT, KO, and DKO groups. **(G)** Heat map showing distribution of intestinal microbiota at genus level from 7-week-old WT, *Bmi-1*^–/–^, and *Bmi-1*^–/–^*p16*^–/–^ mice; the phylum of each genus is listed in the note, *n* = 8. The heat map is colored based on row *Z* scores. The mice with the highest and lowest bacterial levels are in red and blue, respectively.

To investigate if *p16* deletion could ameliorate the dysbiosis in the species and abundance of intestinal microbiota in *Bmi-1^–/–^* mice, principal coordinates analysis of microbial DNA encoding 16S ribosomal RNA (rRNA) was used to detect intestinal microbiota changes. The analysis was carried out at the level of the phylum. Differences were observed between WT and *Bmi-1^–/–^* mice, and the abundance of Bacteroidetes decreased in *Bmi-1*^–/–^ mice when compared with those in WT mice, leading to intestinal microbiota ratio imbalance (Figures 7A,B). *P16* deletion obviously decreased the abundance of Firmicutes and Proteobacteria and increased the abundance of Bacteroidetes in *Bmi-1*^–/–^ mice. After validating that the sequencing sample size meets the requirements of diversity (Figures 7C,D), α-diversity was then assessed to reflect within-sample diversity. The richness of species at the phylum level was lower in the *Bmi-1*^–/–^ group than that in the WT group (Figure 7E). Venn diagram displayed 329 same operational taxonomic units (OTUs) share between the WT and DKO groups, whereas there were only 110 same OTUs between the WT and KO groups (Figure 7F). The results indicated that *p16* deletion could rescue intestinal microbial dysbiosis induced by *Bmi-1* deficiency.

To further clarify the specific changes of intestinal microbiota among *Bmi-1*^–/–^, *Bmi-1*^–/–^*p16*^–/–^, and WT mice, bioinformatics analysis was conducted. Compared with WT mice, *Bmi-1*^–/–^ mice had less diversity of intestinal dominant microbiota, most of which affiliated to Firmicutes including Anaerobtruncus, Lachnospiraceae, Roseburia, and Ruminiclostridium, together with Blautia and Ruminococcaceae, which were closely related to intestinal inflammation. Moreover, the abundance of some intestinal harmful bacteria including *Desulfovibrio*, *Helicobacter*, and *Oscillibacter* was at a higher level in *Bmi-1*^–/–^ mice, whereas the abundance of probiotics such as *Bifidobacterium* was at a lower level. In comparison with *Bmi-1*^–/–^ mice, the abundance of the intestinal harmful bacteria such as *Desulfovibrio* and *Helicobacter* was at a lower level, whereas the diversity of dominant genus elevated, and the abundance of intestinal probiotics including *Bifidobacterium* and *Lactobacillus* was at a higher level in *Bmi-1*^–/–^*p16*^–/–^ mice (Figure 7G). These results demonstrated that *p16* deletion ameliorated intestinal microbial dysbiosis including increasing intestinal microbiota diversity and probiotic abundance and decreasing the abundance of harmful microbiota in *Bmi-1*^–/–^ mice.

### Intestinal Microbial Function Ameliorated by *p16* Deletion in *Bmi-1^–/–^* Mice

**FIGURE 8:**
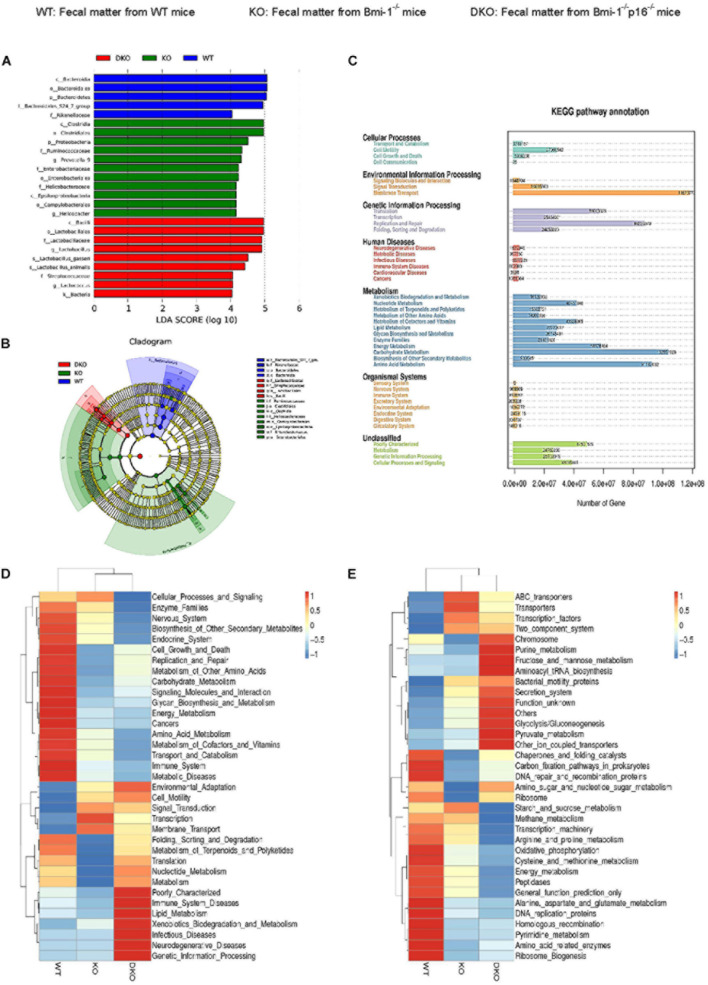
*P16* deletion improved intestinal microbial function ameliorated in *Bmi-1^–/–^* mice. High-throughput sequencing of V4 region amplicon of 16S rRNA from intestinal microbiota was conducted using the fecal of 7-week-old WT, *Bmi-1*^–/–^, and *Bmi-1*^–/–^*p16*^–/–^ mice. **(A)** LEfSe results represented significant differences in bacterial taxa enriched in the WT, KO, and DKO groups. Only taxa with LDA > 4.0 are shown. **(B)** The evolutionary branch map obtained from LEfSe sequence of WT, *Bmi-1*^–/–^, and *Bmi-1*^–/–^*p16*^–/–^ mice. Radiation circle represents the classification level from phylum to genus. Yellow circle indicates no difference; red, green, and blue represent species with significant difference in each group. **(C)** Functional enrichment analysis of KEGG pathway. **(D,E)** Heat map of functional analysis based on the average abundance of KEGG pathway in the three groups, *n* = 8. The heat map is colored based on row *Z* scores. The mice with the highest and lowest functional enrichment are in red and blue, respectively.

To determine if *p16* deletion could ameliorate intestinal microbial function in *Bmi-1* mice, Phylogenetic Investigation of Communities by Reconstruction of Unobserved States (PICRUST) sequencing analysis was carried out based on principal coordinates analysis of microbial DNA encoding 16S rRNA. Linear discriminant analysis (LDA) sequence was used to make clear species with significant differences among samples. The results illustrated that there were 5, 11, and 9 bacterial biomarkers, respectively, in the intestinal microbiota of WT, *Bmi-1*^–/–^, and *Bmi-1*^–/–^*p16*^–/–^ mice (Figure 8A). Distribution of marker bacteria in three genotypes of mice is shown in the cladogram (Figure 8B). These results could help understand the biomarkers of intestinal microbial changes in WT, *Bmi-1*^–/–^, and *Bmi-1*^–/–^*p16*^–/–^ mice.

To further analyze the function of intestinal microbiota, we carried out the PICRUST sequencing analysis and functional enrichment analysis of Kyoto Encyclopedia of Genes and Genomes (KEGG) pathway on the microbiota with significant difference between WT and *Bmi-1*^–/–^ groups. The results showed that the number of genes related to human functional diseases, including 502,983 functional genes related to human immune system diseases, 3,638 functional genes related to cardiovascular disease, and 148,016 functional genes related to the circulatory system. Environmental information processing, genetic information processing, and metabolism were found as the three aspects with the highest gene abundance (Figure 8C).

Function prediction heat map was drawn based on the functional annotation and abundance information of all intestinal microbial samples in KEGG, showing the top 35 bacteria in abundance ranking. An obvious decline was shown in intestinal microbiota energy metabolism; cell growth and death regulation; replication and repair; carbohydrate, glycan, and nucleotide metabolism; terpenoid and polyketide metabolism; protein folding sequencing degradation; and translation function in *Bmi-1*^–/–^ mice compared with WT mice. Among them, the metabolic function mainly focuses on cell motility, membrane transport, and transcription. In comparison with *Bmi-1*^–/–^ mice, the lipid and xenobiotics metabolism functions were enhanced in *Bmi-1*^–/–^*p16*^–/–^ mice (Figure 8D).

Moreover, the genetic information function of intestinal microbiota was predicted and analyzed. In *Bmi-1*^–/–^ mice compared with WT mice, a significant decrease was shown in recombination proteins, peptidase, DNA replication proteins, and amino acid–related enzymes of the intestine; a significant decline was shown in DNA repair, the function of amino sugar and nucleic acid sugar metabolism, cystine and methionine metabolism, pyrimidine metabolism, and oxidative phosphorylation. The function of purine metabolism, fructose, and mannose metabolism, pyruvate metabolism, aminoacyl tRNA biosynthesis, secretion system, and glycolysis were enhanced in intestinal microbiota of *Bmi-1*^–/–^*p16*^–/–^ mice compared with that of *Bmi-1*^–/–^ mice (Figure 8E).

These results demonstrated that metabolism and biosynthesis of intestinal microbiota were ameliorated by *p16* deletion in *Bmi-1*^–/–^ mice. It suggested that the disorder and dysfunction of intestinal microbiota mentioned above might help explain how *Bmi-1*^–/–^ mice developed premature aging and aging-related diseases.

### *Desulfovibrio* Promoting TNF-α Secretion and NF-κB Signaling Activation in Macrophages Ameliorated by *p16* Deletion in *Bmi-1^–/–^* Mice

**FIGURE 9:**
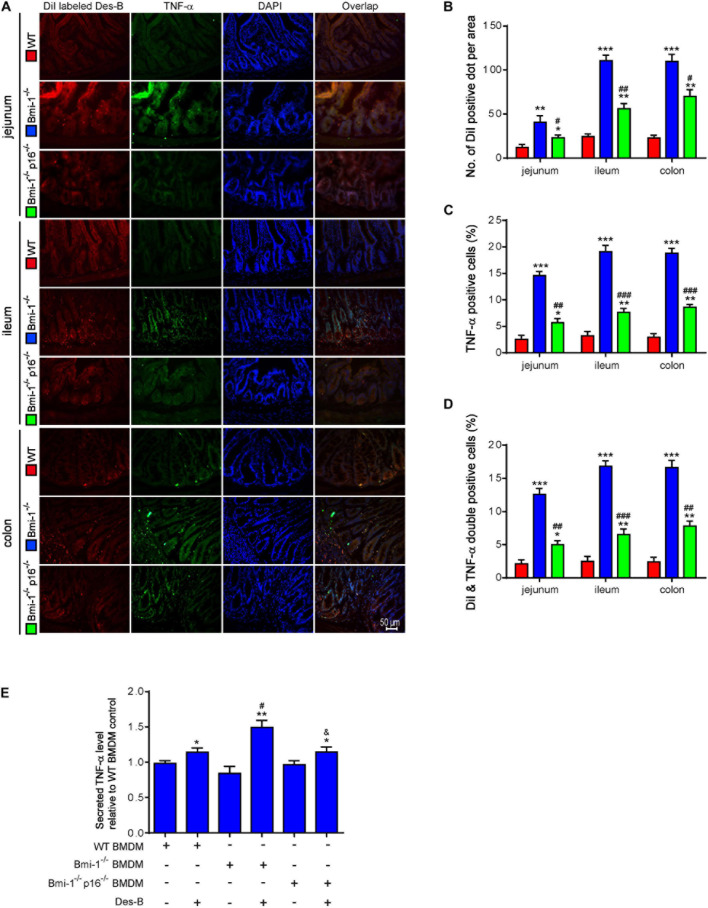
*P16* deletion ameliorated the increase in macrophages-secreted TNF-α resulting from *Bmi-1* deficiency. DiI-labeled *Desulfovibrio* (Des-B) was administered by oral gavage into 7-week-old *Bmi-1*^–/–^, *Bmi-1*^–/–^*p16*^–/–^, and WT mice. **(A)** Representative micrographs showing immunofluorescence for TNF-α in jejunum, ileum, and colon, with DAPI staining the nuclei. **(B)** Number of DiI-positive dot per area. **(C)** TNF-α–positive cells. **(D)** Dil-labeled Des-B and TNF-α double-positive cells. Six mice per group were used for experiments. Values are mean ± SEM from six determinations per group, *p < 0.05, **p < 0.01, ***p < 0.001 compared with the WT group; ^#^*p* < 0.05, ^##^*p* < 0.01, ^###^*p* < 0.001 compared with the *Bmi-1*^–/–^ group. BMDMs was isolated and cultured from bone marrow of WT, *Bmi-1*^–/–^, and *Bmi-1*^–/–^*p16*^–/–^ mice. **(E)** Secreted TNF-α level was detected in the supernatant of BMDMs treated with Des-B or vehicle using ELISA. Cell experiments were performed with three biological repetitions per group. Statistical analysis was performed with one-way ANOVA test. Values are mean ± SEM from six determinations per group, *p < 0.05, **p < 0.01 compared with the Des-B vehicle group; ^#^*p* < 0.05, ^##^*p* < 0.01, ^###^*p* < 0.001 compared with the same treatment of WT-BMDM group; ^&^*p* < 0.05 compared with the same treatment of *Bmi-1^–/–^*–BMDMs.

To determine if *p16* deletion ameliorated the effect of *Desulfovibrio* on promoting TNF-α secretion in intestines of *Bmi-1*^–/–^ mice, DiI-labeled *Desulfovibrio* was administered by oral gavage into *Bmi-1*^–/–^, *Bmi-1*^–/–^*p16*^–/–^, and WT mice. The number of DiI-positive dots per area, TNF-α–positive cells, and DiI- and TNF-α double-positive cells were analyzed after immunofluorescence staining for TNF-α in intestines. In *Bmi-1*^–/–^ mice compared with WT mice, a significant increase was shown in the number of DiI-positive dots per area, TNF-α–positive cells, and DiI and TNF-α double-positive cells in jejunum, ileum, and colon; however, they were significantly reduced in *Bmi-1^–/–^p16^–/–^* mice compared with the *Bmi-1^–/–^* mice (Figures 9A–D). These results demonstrated that more *Desulfovibrio* entered the intestinal epithelium and produced lots of TNF-α in *Bmi-1*^–/–^ mice, which also confirmed the serious destruction of epithelial barrier for this mouse.

To further investigate if *p16* deletion ameliorated the effect of *Desulfovibrio* on promoting macrophages-secreted TNF-α in *Bmi-1*^–/–^ mice, *Desulfovibrio* or vehicle was administered to BMDMs of *Bmi-1*^–/–^, *Bmi-1*^–/–^*p16*^–/–^, and WT mice. Secreted TNF-α level was detected in the supernatant of BMDMs with enzyme-linked immunosorbent assay (ELISA). Results showed that an obvious increase was observed in secreted TNF-α level in *Desulfovibrio*-treated BMDMs compared with vehicle-treated BMDMs at the same genotype. After *Desulfovibrio* treatment, the elevation of secreted TNF-α level was obviously more than WT BMDMs in *Bmi-1*^–/–^ BMDMs; however, it was significantly reduced in *Bmi-1^–/–^p16^–/–^* BMDMs compared with the *Bmi-1^–/–^* BMDMs (Figure 9E).

**FIGURE 10:**
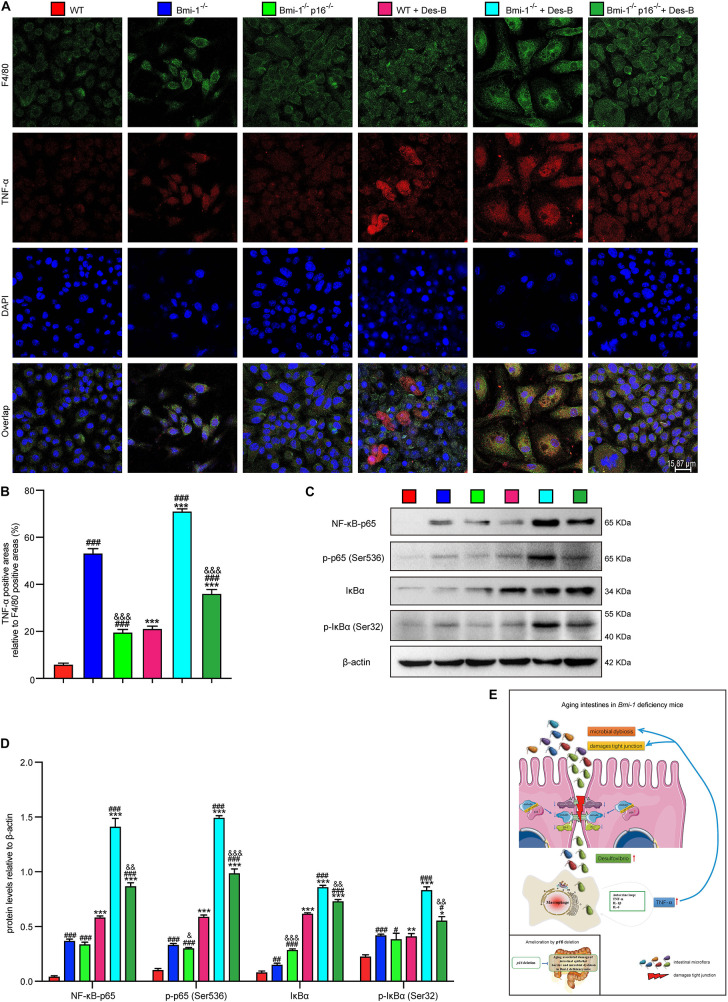
*Desulfovibrio* promoted the TNF-α secretion and activated the NF-κB signaling in BMDMs. BMDMs from WT, *Bmi-1*^–/–^, and *Bmi-1^–/–^p16^–/–^* mice were pretreated with *Desulfovibrio* (10 μg/mL) for 12 h. **(A)** Representative micrographs showing immunofluorescence for F4/80 and TNF-α, with DAPI staining the nuclei. **(B)** Percentage of TNF-α–positive areas relative to F4/80 (%). **(C)** Western blots for NF-κB–p65, p-p65 (Ser536), IκB-α, and p-IκBα (Ser32) in BMDM cells from WT, *Bmi-1*^–/–^, and *Bmi-1^–/–^p16^–/–^* mice; β-actin was used as the loading control. **(D)** Protein levels relative to β-actin were assessed by densitometric analysis. Statistical analysis was performed with one-way ANOVA test. Values are mean ± SEM from six determinations per group, ^∗^*p* < 0.05, ^∗∗^*p* < 0.01, ^∗∗∗^*p* < 0.001 compared with the same genotype without *Desulfovibrio* treatment group; ^#^*p* < 0.05, ^##^*p* < 0.01, ^###^*p* < 0.001 compared with WT-BMDM group at the same treatment; ^&^*p* < 0.05, ^&&^*p* < 0.01, ^&&&^*p* < 0.001 compared with the *Bmi-1^–/–^*–BMDM group at the same treatment. **(E)** Graphical abstract about aging intestines in *Bmi-1*–deficient mice. Macrophages-secreted TNF-α resulting from *Bmi-1* deficiency not only stimulated macrophages to secrete more inflammatory factors, but also accelerated the intestinal microbiota disorders and destruction of TJ. Harmful bacteria including *Desulfovibrio* cross TJ to further trigger systemic inflammations. Up-regulated p16 in the intestine of *Bmi-1*–deficient mice prevented occludin from forming and repairing intercellular TJ by combining with occludin protein at the 1st–160th residue in the cytoplasm, which exacerbated destruction of TJ. However, *p16* deletion improved intestinal microbial function and maintained the intestinal epithelial barrier in *Bmi-1*–deficient mice.

To further investigate if *Desulfovibrio* promoted the TNF-α secretion and activated the NF-κB signaling in BMDMs, primary BMDM cells from WT, *Bmi-1*^–/–^, and *Bmi-1^–/–^p16^–/–^* mice were treated with *Desulfovibrio* (10 μg/mL for 12 h). Immunofluorescence staining was also used to observe the expression level of TNF-α. Western blot was used to detect the expression levels of NF-κB-p65, p-p65 (Ser536), IκBα, and p-IκBα (Ser32). Our results showed that *Bmi-1* deficiency caused BMDMs to increase the secretion of TNF-α, and the expressions of NF-κB-p65, p-p65 (Ser536), IκBα, and p-IκBα (Ser32); however, *p16* deletion partially corrected this process (Figures 10A–D). *Desulfovibrio* treatment promoted the secretion of TNF-α (Figures 10A,B), and the expressions of NF-κB-p65, p-p65 (Ser536), IκBα, and p-IκBα (Ser32) in WT, *Bmi-1*^–/–^, and *Bmi-1^–/–^p16^–/–^* BMDMs compared with the same genotype BMDMs without *Desulfovibrio* treatment (Figures 10C,D).

These results demonstrated that *Desulfovibrio* promoted the TNF-α secretion and activated the NF-κB signaling in BMDMs, and *p16* deletion ameliorated the effect of *Desulfovibrio* on promoting macrophages-secreted TNF-α in *Bmi-1*^–/–^ mice.

## Discussion

This study demonstrated that in an SIPS model of *Bmi-1* deficiency, barrier structure and function in intestinal epithelium were damaged; TNF-α–induced destruction of TJ increased barrier permeability; up-regulated proinflammatory level of macrophages was induced by intestinal microbial dysbiosis and dysfunction; and more *Desulfovibrio* entered the intestinal epithelium to promote TNF-α secretion and NF-κB signaling activation in macrophages. P16 protein was accumulated in aging intestinal epithelium from *Bmi-1*–deficient mice, combined with occludin in the cytoplasm, and finally prevented the form and repair of TJ. *P16* deletion ameliorated damage of intestinal epithelial barrier and microbial dysbiosis caused by *Bmi-1* deficiency. FM transplantation from WT mice ameliorates TJ in intestinal epithelium of *Bmi-1* deficiency and *Bmi-1* and *p16* double-deficient mice (Figure 10E).

Bmi-1 is a member of the polycomb repressor complex 1 that mediates gene silencing by regulating chromatin structure and is indispensable for self-renewal of both normal and cancer stem cells (Bhattacharya et al., 2015). It has been reported that Bmi-1 also plays a crucial role in maintaining self-renewal of ISCs in the crypt of epithelium. When the stem cells responsible for daily maintenance of intestinal epithelium are destroyed, *Bmi-1–*expressing cells increase in number, acting as a compensatory mechanism (Tian et al., 2011). Several lines of evidence demonstrate that aging leads to the dysfunction of ISCs in intestinal crypts, driving rapid renewal of intestinal epithelium, generating progenitor cells, and differentiating several cell types such as Paneth cells and goblet cells, which are critical to maintain intestinal epithelial function and homeostasis (Henning and von Furstenberg, 2016; Moorefield et al., 2017). Our previous reports demonstrate that *Bmi-1–*deficient mouse is an SIPS model, induced by imbalanced redox and DNA damage (Zhang et al., 2010; Jin et al., 2014, 2017; Xie et al., 2015; Chen et al., 2020; Sun et al., 2020). We observed that besides the smaller body size and shorter intestinal length, *Bmi-1*–deficient mice also showed abnormal intestinal morphology, such as shorter villus and down-regulation of glycoprotein and mucin, which made up a relatively significant share of the intestinal mucus. Length of villi influences the absorptive surface involved in digestion (Rao and Fritz-Niggli, 1988). Mucus in small intestine is important for efficient nutritional uptake, whereas the mucus in colon helps keep bacteria away from the epithelium (Johansson and Hansson, 2016). In this study, our results showed TJ destruction in intestinal epithelial cells from *Bmi-1*–null mice. TJ integrity, closely associated with intestinal epithelium function, contributes to the function of physical intestinal barrier by regulating paracellular transportation (Lee et al., 2018). Previous report finds that intestinal *Bmi-1* deficiency inhibits epithelium proliferation and hampers self-renewal of the ISCs accompanied by p16 accumulation (Lopez-Arribillaga et al., 2015). In this study, we found that aging epithelium cells characterized by p16 accumulation inhibited proliferation and hampered repair of intestinal epithelium and further disrupted microbial homeostasis. Moreover, the up-regulated proinflammatory level of intestinal macrophages was associated with the change of intestinal microbiota in *Bmi-1*–deficient mice. *Bmi-1* deficiency resulted in significant decreases in the body weight, villus length, the ratio of villus length to crypt depth, the number of Paneth cells, and the number of acid mucin and glycoprotein. Thus, we speculated that all changes of the intestine caused by *Bmi-1* deficiency weakened intestinal immune barrier function, nutrient absorption, and intestinal immunity in mice, which finally contributed to the smaller body size and increased inflammation level of mice.

TJ barrier of the intestine is formed by claudins, occludin, and ZO-1, helping to maintain the selective barrier function of intestinal epithelium (Lee et al., 2018). Among these, occludin is important in the assembly and maintenance of TJ (Raleigh et al., 2011). Occludin that is phosphorylated locates mainly in the membrane, while the less phosphorylated occludin is found in the cytoplasm (Lee et al., 2018). In our study, we observed breakage of cell–cell TJ in the intestinal epithelium of *Bmi-1*–deficient mice, locating exactly at the occludin section. Also, the occludin is down-regulated in intestinal epithelium of *Bmi-1*–deficient mice. These conditions were then reversed by *p16* deletion. We then investigated how *p16* deletion rescues intestinal epithelial barrier dysfunction. Former experiments discovered that N-terminal of p16 (residues 1–80) bound to the N-terminal region of JNK1 (also known as MAPK8) (residues 1–60) or the N-terminal region of JNK3 (also known as MAPK10) (residues 75–100), which contain the glycine-rich site (Choi et al., 2005). Analysis with https://www.uniprot.org/align showed an extremely similar domain between occludin (residues 107–139, which is a glycine-rich site) and MAPK8 (residues 16–38) or MAPK10 (residues 54–76) in human. In mice, an extremely similar domain showed between occludin (residues 107–139) and MAPK8 (residues 24–72) or MAPK10 (residues 62–110). Besides, the 107th-139th residue is also a glycine-rich site of occludin for mouse. As a transmembrane protein, the 90th-135th residue of occludin locates at the extracellular domain. Thus, we speculated that p16 could bind with occludin in cytoplasm, blocking occludin in participating in the formation of intercellular TJ, which further aggravated the destruction of TJ in *Bmi-1*–deficient mice. Then, we found that p16 and occludin were colocalized and combined in the cytoplasm of intestinal epithelial cells. Accumulated p16 combined with occludin at the 1st–160th residue rather than limited to the 107th–139th residue in the cytoplasm of aging intestinal epithelium cells in *Bmi-1*^–/–^ mice, inhibiting its translocation from cytoplasm to cell membrane for blocking the TJ formation and repair of intestinal epithelium barrier. Moreover, the premature senescence induced by *Bmi-1* deficiency caused destruction of intestinal TJ by TNF-α and reduced levels of TJ proteins, exacerbating the destruction of intercellular TJ and increasing intestinal epithelial permeability.

Several lines of evidence demonstrate that TNF-α stimulates permeability and disrupts integrity by up-regulating myosin light chain kinase (MLCK) aggravating intestinal epithelial barrier dysfunction and immune-mediated colitis, and/or reduces TJ protein expressions through activating NF-κB signaling pathway via TNFR1 and ERK1/2 signaling pathway (Ye et al., 2011; Hall et al., 2017; Drolia et al., 2018). Our results confirmed that TNF-α treatment damaged TJ and decreased the expressions of occludin and ZO-1. It has been reported that TNFR2 plays an important role in stimulating epithelial long MLCK expression, aggravating intestinal epithelial barrier dysfunction and immune-mediated colitis. TNFR2 interferes with programmed cell death by activating NF-κB and JNK pathways or by inhibiting TRAF-2 (Su et al., 2009, 2013). In this study, we detected TNF-α and other related proinflammatory factor levels in ileum, jejunum, and colon from *Bmi-1*–deficient mice and observed the up-regulation of proinflammatory factor and activation of NF-κB pathway. Besides, we observed no difference in phagocytosis of macrophage between *Bmi-1* deficiency and WT mice. However, how Bmi-1 and/or p16 regulates the subtype and function of macrophages remains unknown. Previous observation finds that the expression of p16 and senescence-associated β-galactosidase (SA-β-gal) are markers of their physiological programs of polarization in response to immunomodulatory stimuli in macrophages. This characterization is reversible and p53-independent in macrophages and obviously different from cellular senescence characterized by the constitutive expression of these biomarkers following p53-dependent proliferation arrest in rodent cells at least. Thus, p16 and/or SA-β-gal–positive phases are non-senescent phases in macrophages (Hall et al., 2017). In this study, BMDMs in *Bmi-1*–null conditions, especially in *Bmi-1*–null plus *Desulfovibrio* treatment, showed a bigger size and irregular shapes. We think that this shape might be determined by its physiological programs of polarization in response to immunomodulatory stimuli in macrophages, although not because they are senescent. We will investigate whether Bmi-1 determines the polarization and response of macrophages through inhibiting p16 in the follow-up study. It has been reported that chronic exposure to inflammation alters proinflammatory function of macrophage, thus secreting more proinflammatory factors (Thevaranjan et al., 2017). In this study, we found that the up-regulated proinflammatory level of macrophages was associated with the change of aging associated intestinal microbiota in *Bmi-1*–null mice.

A former study has reported that intestinal microbiota dysbiosis not only triggers inflammatory bowel disease (Larabi et al., 2020), but also leads to systemic inflammation, obesity, type 2 diabetes, and chronic kidney disease (Sabatino et al., 2017; Thevaranjan et al., 2017; Virtue et al., 2019). Thus, we conducted high-throughput sequencing of V4 region amplicon of 16S rRNA to detect intestinal microbiota changes, which showed the intestinal microbiota ratio imbalance. Besides the diversity of intestinal bacterium, the number of intestinal probiotics also decreased after *Bmi-1* deletion, whereas the pathogenic bacteria such as *Desulfovibrio*, *Helicobacter*, and *Oscillibacter* increased, leading to the dysbiosis of microbiota. Moreover, the function of carbohydrate, glycan and nucleotide metabolism, cell growth and death regulation, replication, and repair were all impaired in *Bmi-1*–deficient mice, which further damaged the intestinal function and nutrition absorption. Not only the pathogenic bacteria itself, but also the harmful substances it secreted can also lead to intestinal diseases and even systemic diseases (Moreto and Perez-Bosque, 2009). Then, DiI-labeled *Desulfovibrio*, a kind of pathogenic bacteria, was administered by oral gavage into *Bmi-1* knockout, *Bmi-1* and *p16* double-knockout, and WT mice. TNF-α level and the number of *Desulfovibrio* were increased in the intestinal epithelium of *Bmi-1*–deficient mice. To further confirm whether up-regulation of TNF-α is secreted by macrophages through activating NF-κB signaling after *Desulfovibrio* inducing, TNF-α protein level was up-regulated in the supernatant, and NF-κB signaling was activated in BMDMs from the mice, illustrating that *Desulfovibrio* could induce macrophages secreting more TNF-α and further aggravate the damage of intestinal barrier. We also observed that *p16* deletion could ameliorate the microbiota dysbiosis and decrease the inflammation level in *Bmi-1*–deficient mice, which provides potential targets for the treatment of age-associated intestinal diseases and intestinal microbiota dysfunction.

Taken together, our results demonstrated that Bmi-1 maintained intestinal TJ, epithelial barrier function, and microbiota balance through preventing senescence characterized by p16 accumulation. Clearance of p16-positive cells in aging intestinal epithelium would be a new method for maintaining barrier function and microbiota balance. The 1st to 160th residues of occludin could be a novel therapeutic target for identifying small molecular antagonistic peptides to prevent the interaction of p16 with occludin for protecting TJ.

## Materials and Methods

### Mice and Genotyping

*Bmi-1^–/–^p16^–/–^*, *Bmi-1^–/–^*, and WT SPF mice were prepared as described previously (Jin et al., 2014, 2017). All of them were housed in pathogen-free conditions. For studies of the microbiota, mice were selected from multiple cages and multiple breeding pairs to minimize cage effects or familial transfer of the microbiota as previously described (Thevaranjan et al., 2017). No evidence of cage effects was found in the studies of the microbiota. This study was carried out in strict accordance with the guidelines of the Institute for Laboratory Animal Research of Nanjing Medical University in Nanjing of China. The protocol was approved by the Committee on the Ethics of Animal Experiments of Nanjing Medical University (permit number IACUC-1706001).

### Cell Cultures

Seven-week-old mice were anesthetized with 3% pentobarbital sodium (40 mg/kg). The leg bones were separated, and bone marrow was rushed out with phosphate-buffered saline (PBS). BMDMs were then isolated and cultured in Dulbecco modified eagle medium (DMEM) supplemented with 10% fetal bovine serum (FBS) (Gibco, Life Technologies Inc., NY, United States) and 50 ng/mL recombinant mouse macrophage colony-stimulating factor (Novoprotein Scientific Inc., Shanghai, China) at 37°C as previously described (Assouvie et al., 2018).

The Cacao-2 cells (#ZQ0056) (Shanghai Zhong Qiao Xin Zhou Biotechnology Co., Ltd., Shanghai, China) were cultured in 1640 medium with 10% FBS according to the manufacturer’s instructions.

### Preparation of Fecal Microbiota

Feces were collected daily from 3- to 4-week-old WT or *Bmi-1*^–/–^ mice in sterile conditions. Stools from each group were pooled 100 mg and then resuspended in 1 mL of sterile saline. The solution of FM was vigorously mixed before centrifugation at 800 *g* for 3 min as previously described (Chang et al., 2015). The supernatant bacterial concentration of FM was detected with microplate reader at 600-nm excitation wavelength and should be more than 0.6; 0.6–0.8 is the logarithmic phase of bacterial growth, and 0.8–1.0 is the platform phase of bacterial growth. The concentration of FM was used in both phases.

The supernatant from the WT group was collected and labeled with DiI (Sigma-Aldrich, St. Louis, MO, United States) for tracking according to the manufacturer’s protocol before infecting BMDMs as previously described methods (Zhang et al., 2011; Nagyova et al., 2014; Xie et al., 2015). Then, the DiI-labeled FM was incubated with BMDMs for immunofluorescent staining of cells. The supernatant from WT or *Bmi-1*^–/–^ group was collected and incubated with BMDMs for real-time reverse transcriptase–polymerase chain reaction (RT-PCR) detection.

### Fecal Microbiota Transplantation

Mice were deprived of food 4 h before an oral gavage. FM from 3- to 4-week-old WT mice (fecal samples 100 μL, 100 mg/mL) were transplanted to WT, *Bmi-1*^–/–^, and *Bmi-1*^–/–^*p16*^–/–^ mice by gavage every other day and lasted for 21 days.

### Preparation of the Intestine Sections

Mice were anesthetized with 3% pentobarbital sodium (40 mg/kg) at 7 weeks of age. Jejunum, ileum, and colon were separated and washed with PBS. The intestines were cut into several pieces following the annular surface. Then, the intestine samples were fixed with periodate-lysine-paraformaldehyde (PLP) solution for 24 h at 4°C (Zhang et al., 2010). For hematoxylin–eosin (H&E), Alcian blue and periodic acid–Schiff (AB-PAS) staining, immunohistochemistry, and immunofluorescence, the samples were dehydrated in a series of graded ethanol solutions, embedded in paraffin, and cut into 5-μm sections using a rotary microtome (Leica Biosystems Nussloch GmbH, Nussloch, Germany) as previously described (Jin et al., 2017). For immunofluorescence staining, the samples were dehydrated in 15 and 30% sucrose solutions (prepared in 1 × PBS) in turn until the tissue settled to the bottom of the tube. Then, the samples were cut into 5-μm sections using a freezing microtome (Thermo Scientific Cryotome FSE Cryostats, Loughborough, Leicestershire, England) as previously described (Chen et al., 2020).

### Histology Staining

For H&E, AB-PAS staining, or immunofluorescence, serial paraffin sections were deparaffinized and rehydrated.

### Hematoxylin–Eosin Staining

Serial paraffin sections of jejunum, ileum, and colon were stained as previously described (Jin et al., 2017).

### Alcian Blue and Periodic Acid–Schiff Staining

AB-PAS staining kit (#G1285, Solarbio Life Sciences, Beijing, China) was used according to the manufacturer’s instructions and following methods as previously described (Li et al., 2019).

### Immunohistochemical Staining

Serial paraffin sections were generated for antigen retrieval, steamed for 20 min in sodium citrate buffer (10 mM sodium citrate acid, 0.05% Tween-20, pH 6.0) followed by blocking of endogenous peroxidase (3% H_2_O_2_) and preincubation with serum (Jin et al., 2014; Xie et al., 2015). Primary antibodies were against Bmi-1 (#5856, Cell Signaling Technology, United States), p16 (#ab211542, Abcam, Cambridge, MA, United States), p53 (#2524, Cell Signaling Technology, United States), and Ki67 (#BS1679, Bioworld Technology Inc., MN, United States). Biotin-labeled second antibody was used. Nuclei were stained with hematoxylin.

### Immunofluorescent Staining

Primary antibodies against ZO-1 (sc33725, Santa Cruz Biotechnology Inc., Dallas, TX, United States), TNF-α (sc-52746, Santa Cruz Biotechnology Inc., United States), F4/80 (sc-377009, Santa Cruz Biotechnology Inc., United States), occludin (sc133256, Santa Cruz Biotechnology Inc., United States), and p16 (10883-1-AP, Proteintech Group, Inc., IL, United States) were used, and affinity-purified Alexa Fluor 488–conjugated secondary antibody and 594-conjugated secondary antibody (Life Technologies Corporation, Carlsbad, CA, United States) were used. Nuclei were labeled with DAPI (Sigma-Aldrich, United States) and mounted with medium to prevent quenching (Vector Laboratories Inc., United States) following previously described methods (Jin et al., 2017).

### Construction and Transfection of Overexpressed Adenovirus or Plasmid of Full-Length, Mutant, or Truncated Fragments

*GFP-labeled p16* full-length overexpression adenovirus carrying the Flag-tag was designed and synthesized by Genechem Co., Ltd., in Shanghai, China.

Considering the structural features of occludin as a transmembrane protein, we generated four mutant plasmids based on its intracellular and extracellular domain, which contains mutation of 1–160 residues, 161–265 residues, 266–522 residues, and 107–139 residues and a full-length or a truncated fragment (residues 266–522) of occludin overexpression plasmid all carrying the His-tag with pcDNA3.1 vector plasmid. Production of the above plasmids was by TranSheep Bio Co., Ltd., Shanghai, China.

The above adenovirus was transfected in 293T cells incubated with antibiotic-free complete medium at 40–50% fluence for 8 h. After 24 h, plasmids were transfected into these 293T cells using X-tremeGENE HP DNA Transfection Reagent (cat. no.06366236001, Roche Diagnostics GmbH, Mannheim, Germany). Cells were incubated with antibiotic-free complete medium including the above transfection mixture for 48 h, and protein was harvested. Specific details followed the instruction of X-tremeGENE HP DNA Transfection Reagent.

### Immunofluorescent Staining of Cells

When the Cacao-2 cell density reached 60%, they were transfected with GFP-labeled *Flag-p16*–overexpressed (*p16*-OV) or vehicle adenovirus and then treated with or without 10 ng/mL TNF-α (#C008, Novoprotein Scientific Inc., Shanghai, China) for 24 h. Then, Cacao-2 cells were fixed with PLP for 10 min and washed three times with PBST (PBS with 0.05–0.1% Tween-20) for 10 min each and blocked with 5% bovine serum albumin at room temperature for 1 h to prevent non-specific reactions. Primary antibodies against F4/80 (sc-377009, Santa Cruz Biotechnology Inc., United States), ZO-1 (#21773-1-AP, Proteintech Group, Inc., IL, United States), and occludin (sc133256, Santa Cruz Biotechnology Inc., United States) were used. Affinity-purified Alexa Fluor 488–conjugated secondary antibody (Life Technologies Corporation, United States) was used. Nuclei were labeled with DAPI.

BMDMs were pretreated with *Desulfovibrio* (10 μg/mL) for 12 h or cultured with DiI-labeled FM preincubated with serum. Cells were reacted with primary antibody after being fixed and blocked. Primary antibodies against F4/80 (sc-377009, Santa Cruz Biotechnology Inc., United States) and TNF-α (NBP1-19532, Novus Biologicals, Centennial, Co., United States) were used, and affinity-purified Alexa Fluor 488–conjugated secondary antibody (Life Technologies Corporation, United States) was used. Nuclei were labeled with DAPI (Sigma-Aldrich, United States) and mounted with medium to prevent quenching (Vector Laboratories Inc., Burlingame, CA, United States) (Gu et al., 2016).

### Duolink Proximity Ligation Assay

After routinely dewaxing and hydration, serial paraffin sections of ileum were blocked with sheep serum and detected with antibodies against p16 (#ab211542, Abcam, Cambridge, MA, United States) and occludin (#sc-133256, Santa Cruz Biotechnology Inc., United States). Then, Duolink Proximity Ligation Assay (PLA) *in situ* fluorescence (Sigma-Aldrich, United States) was performed following the manufacturer’s instructions with Duolink *in situ* PLA probe anti-mouse PLUS (#DUO92001), Duolink *in situ* PLA probe anti-rabbit MINUS (#DUO92005), Duolink *in situ* detection reagents Red (#DUO92008), and Duolink *in situ* wash buffer fluorescence (#DUO82049). The PLA signal (λex 594 nm, λem 624 nm; Texas Red) was analyzed (Chen et al., 2020).

### Transmission Electron Microscopy

Intestinal samples of mice were fixed with 1% glutaraldehyde in a 0.1 M sodium phosphate buffer (pH 7.4, 4°C, 2 h), postfixed in 2% osmium tetroxide in a 0.1 M phosphate buffer (4°C, 1.5 h), and dehydrated in a graded series of concentrations of ethanol (50, 70, 90, 95, and 4 × 100% each for 15 min) and acetone (15 min). Eventually, the material was embedded in epoxy resin. Sections were cut to stain with uranyl acetate and lead citrate (Jin et al., 2014; Wang et al., 2019). JEOL 1200EX TEMSCAN electron microscope was used to observe the TJ of intestinal epithelial cells.

### Intestinal Permeability

Tracer FITC–labeled dextran (#46944; Sigma-Aldrich) was used to assess *in vivo* intestinal permeability as previously described (Thevaranjan et al., 2017). Mice were deprived of food 4 h before an oral gavage using FITC–dextran (600 mg/kg body weight, 125 mg/mL). Blood was collected from heart after 6 h, and fluorescence intensity was measured on fluorescence plates using an excitation wavelength of 493 nm and an emission wavelength of 518 nm (Thevaranjan et al., 2017).

### Western Blots

Intestinal epithelium of 7-week-old mice or BMDM cells was dissected and immediately placed into RIPA lysis buffer containing a cocktail of proteinase inhibitors and phosphatase inhibitors (#4906845001, Roche Diagnostics Corp., Basel, Switzerland) and phenylmethanesulfonyl fluoride (#ST506, Beyotime Institute of Biotechnology, Shanghai, China) for protein extraction. Western blots were performed as previously described (Smith et al., 2003; Jin et al., 2017). Primary antibodies against claudin-1 (sc-81796, Santa Cruz Biotechnology Inc., United States), occludin (sc133256, Santa Cruz Biotechnology Inc., United States), claudin-2 (sc-293233, Santa Cruz Biotechnology Inc., United States), TNF-α (NBP1-19532, Novus Biologicals, Centennial, Co., United States), NF-κB–p65 (#8242, Cell Signaling Technology, Beverly, MA, United States), p-p65 (Ser536) (ab76302, Abcam, United States), IκBα (AF1282, Beyotime Biotechnology, China) and p-IκBα (Ser32) (sc-8404, Santa Cruz Biotechnology Inc., United States), ZO-1 (sc33725, Santa Cruz Biotechnology Inc., United States), p16 (#ab211542, Abcam, United States), DYKDDDDK Tag (binds the same epitope as Sigma’s anti-FLAG M2 antibody, #14793, Cell Signaling Technology, United States), and His-Tag (#12698, Cell Signaling Technology, United States) were used. β-Actin (BS6007M, Bioworld Technology, St. Louis Park, MN, United States) was the loading control for the cytoplasmic fraction and total cell protein.

### RNA Extraction and Real Time Reverse Transcriptase–Polymerase Chain Reaction

RNA was extracted from BMDMs untreated or treated with fecal bacteria using TRIzol reagent (#15596, Invitrogen Inc., United States) according to the manufacturer’s protocol. Levels of mRNA in cell samples were quantified by real-time RT-PCR as previously described (Jin et al., 2011; Jin et al., 2017). Primers were in Supplementary Table 1.

### Immunoprecipitation Analysis

After extracting total protein from mouse ileum tissues, immunoprecipitation assay was performed using Pierce Co-Immunoprecipitation (Co-IP) Kit (#26149, Pierce Co-IP Kit, Thermo Fisher Scientific, IL, United States) as the manufacturer instructed. Followed by preclearing lysate with the control agarose resin, tissue proteins were mixed with 1 μg of antibody and then incubated overnight, anti–immunoglobulin G (IgG) antibody as a control. The bound antigens were eluted from the agarose resin using elution buffer. Eluted samples were carried out with sodium dodecyl sulfate–polyacrylamide gel electrophoresis gel electrophoresis. Immunoblotting was carried out as previously described (Chen et al., 2018). Clean-Blot^TM^ IP Detection Reagent (horseradish peroxidase) (#21230, Thermo Fisher Scientific) was used as secondary antibody to eliminate IgG bands. Primary antibodies against p16 (#ab211542, Abcam, United States), occludin (#sc-133256, Santa Cruz Biotechnology Inc., United States), and DYKDDDDK Tag (binds the same epitope as Sigma’s Anti-FLAG M2 antibody, #14793, Cell Signaling Technology, United States) were used. The immunoreactive bands were visualized by ECL chemiluminescence (Amersham Pharmacia Biotech, NJ, United States) and analyzed by the Scion image Beta 4.02 (Scion, National Institutes of Health, Bethesda, MD, United States).

### Protein Sequence Alignment

The amino acid sequences of occludin, MAPK8, and MAPK10 protein from mouse or human were aligned using online tools (Uniprot/Align)^1^ (Supplementary Information 4, 5 alignment of occludin, MAPK8 and MAPK10 in human or mouse).

### Sample Collection and DNA Extraction

Eight mice were selected from each genotype. Mice feces were collected and then stored at −80°C before use. Total genome DNA was extracted from the samples using DNA magnetic bead extraction kit following the manufacturer’s instructions. After extraction, DNA was treated with DNase-free RNase to remove contaminating RNA. DNA concentration and quality were assessed (Pandit et al., 2018; Zhao et al., 2019). Then, the DNA was stored at −20°C for use.

### 16S rRNA Gene-Based Sequencing Analysis

The V4 region of the 16S rRNA gene was amplified with primers and sequenced as previously described (Caporaso et al., 2010, 2012). Amplified DNA was sequenced using QIIME (Caporaso et al., 2010) software package (Quantitative Insights Into Microbial Ecology, V1.7.0).^2^

Raw data were analyzed by Novogene Bioinformatic Technology Co., Ltd., In-house Perl scripts were used to analyze α- (within samples) and β- (among samples) diversity. Sequences with ≥ 97% similarity were assigned to the same OTUs. We pick representative sequences for each OTU and use the RDP classifier (Wang et al., 2007) to annotate taxonomic information for each representative sequence. In order to compute α-diversity, we rarify the OTU table and calculate three metrics: Chao1 estimates the species abundance; Observed Species estimates the amount of unique OTUs found in each sample, and Shannon index. Based on Unifrac distance, principal component analysis was conducted using QIIME to analyze β-diversity (Hamady et al., 2010). Biomarkers were selected with LEfSe (LDA effect size) (Segata et al., 2011). In total, species with significant differences were analyzed using Wilcoxon rank sum test. LDA score was used to assess the impact of significantly different species.

PICRUST was used to predict the metabolic pathways from 16S rRNA gene-based microbiota of the intestine. The predicted functions were then collapsed into hierarchical KEGG pathways using the categorize-by-function step in the PICRUST pipeline (Garcia-Amado et al., 2018). Heat maps of function categories based on KEGG pathways and abundance changes of the differential bacteria were generated by R software.

### Infection Experiments *in vivo* and *in vitro*

*Desulfovibrio* freeze-dried powder (#BNCC173631) was obtained from Beijing Beina Chuanglian Biotechnology Research Institute in Beijing of China. According to the manufacturer’s instructions, certain amount of germ-free calf serum was added into the *Desulfovibrio* freeze-dried powder and mixed. The bacterial solution was dropped into Colombian blood agar plate with triangle coating rod spreading evenly. It was then cultured in anaerobic culture zone at 37°C. After the colony was formed, its shape was confirmed under the microscope. The *Desulfovibrio* colony was inoculated into LB liquid medium and amplified after passaging two to three times (the whole process was completed in anaerobic culture zone). Every 100 μL of bacterial solution was added into 900 μL of 10% glycerin and stored at −20°C for use.

*In vivo*, before the oral gavage, the concentration of *Desulfovibrio* solution was detected with microplate reader at 600-nm excitation wavelength and should be more than 0.6. *Desulfovibrio* solution was labeled with DiI (Sigma-Aldrich, United States) for tracking according to the manufacturer’s protocol as previously described methods (Xie et al., 2015). After depriving of food 4 h, mice were administered 10^8^ DiI-labeled *Desulfovibrio* by an oral gavage. Then, after 6 h, intestine was taken for immunofluorescence.

*In vitro*, for the infection experiment, the *Desulfovibrio* solution (10 μg/mL) was incubated with BMDMs in α-MEM medium supplemented with 15% FBS (Gibco, United States) at 37°C for 12 h.

### Enzyme-Linked Immunosorbent Assay

BMDMs untreated or infected with *Desulfovibrio* bacteria (Des-B) were cultured in DMEM (without phenol red; Gibco) without FBS for 6 h. Supernatants were collected and filtered with MILLEX-GP 0.22-μm filters (Merck Millipore Ltd., Co., Cork, Munster, Ireland) to remove cell debris, concentrated to 1% volume with Amicon Ultra-4 centrifugal ultrafiltration tubes (NMWL 3KDa) (Merck Millipore Ltd., Co.) (Xie et al., 2015; Jin et al., 2017; Chen et al., 2020), and detected the concentrations of mouse-derived TNF-α with ELISA kit (#YFXEM00031, Yifeixue Biotechnology, Nanjing, China).

### Statistical Analysis

All analyses were performed using GraphPad Prism software (version 6.07; GraphPad Software Inc., San Diego, CA, United States) as previously described (Schafer et al., 2017). Measurement data were described as mean ± SEM fold-change over control and analyzed by Student *t*-test and one-way analysis of variance (ANOVA) to compare differences among groups. Qualitative data were described as percentages and analyzed using χ^2^-tests as indicated (Jin et al., 2014, 2017; Chen et al., 2020). *p*-values were two-sided, and differences were considered significant at *p* < 0.05.

As in previously described statistical methods (Zhang et al., 2020), to identify differential abundance of phyla, genera, and species between any two groups, Wilcoxon rank-sum test was used, *p*-value was corrected as false discovery rate with the Benjamini–Hochberg method. *p*-values were considered significant at *p* < 0.05. To identify features (taxa and functional modules) differentially represented between any two groups, differentially abundant taxa or functional modules were selected using the LEfSe. Only taxa with LDA score > 4.0 are shown.

## Data Availability Statement

The datasets presented in this study can be found in online repositories. The names of the repository/repositories and accession number(s) can be found below: http://www.ncbi.nlm.nih.gov/bioproject/704896. The BioProject ID is PRJNA704896.

## Ethics Statement

The animal study was reviewed and approved by the Committee on the Ethics of Animal Experiments of Nanjing Medical University.

## Author Contributions

JJ and DM: conceptualization and funding acquisition. JWZ, CH, HC, ZQ, ZM, JYZ, QW, MC, CX, RW, QL, GZ, DM, and JJ: methodology. JWZ, CH, HC, ZQ, ZM, CX, RW, QL, and JJ: software. JWZ, CH, HC, ZQ, ZM, and JJ: validation. JWZ, CH, HC, ZQ, ZM, JYZ, QW, MC, CX, RW, QL, GZ, and JJ: data analysis. JWZ, JJ, and DM: writing—original draft with help from the other authors. JJ, HC, and DM: writing—review, and editing with help from the other authors. RW, JJ, and DM: project administration and supervision. All authors contributed to the article and approved the submitted version.

## Conflict of Interest

The authors declare that the research was conducted in the absence of any commercial or financial relationships that could be construed as a potential conflict of interest.

## Publisher’s Note

All claims expressed in this article are solely those of the authors and do not necessarily represent those of their affiliated organizations, or those of the publisher, the editors and the reviewers. Any product that may be evaluated in this article, or claim that may be made by its manufacturer, is not guaranteed or endorsed by the publisher.

## Footnotes

1
https://www.uniprot.org/align/

2
http://qiime.org/index.html

## Abbreviations

*Bmi-1* ^–/–^: *Bmi-1*–deficient
*Bmi-1^–/–^p16* ^*INK4a*–/–^: *Bmi-1* and *p16*^*INK4a*^ double-knockout
TJ: tight junction
SIPS: stress-induced premature senescence: WT-FB, fecal mixed bacteria from WT mice
Des-B: *Desulfovibrio* bacteria
ISCs: intestinal stem cells.
