# Supplementary figures and images for: P16INK4a Deletion Ameliorates Damage of Intestinal Epithelial Barrier and Microbial Dysbiosis in a Stress-Induced Premature Senescence Model of Bmi-1 Deficiency
Source: Front Cell Dev Biol. 2021 Oct 7;9:671564. doi: 10.3389/fcell.2021.671564 (PMC8545785; doi:10.3389/fcell.2021.671564)

Figure S1

A.

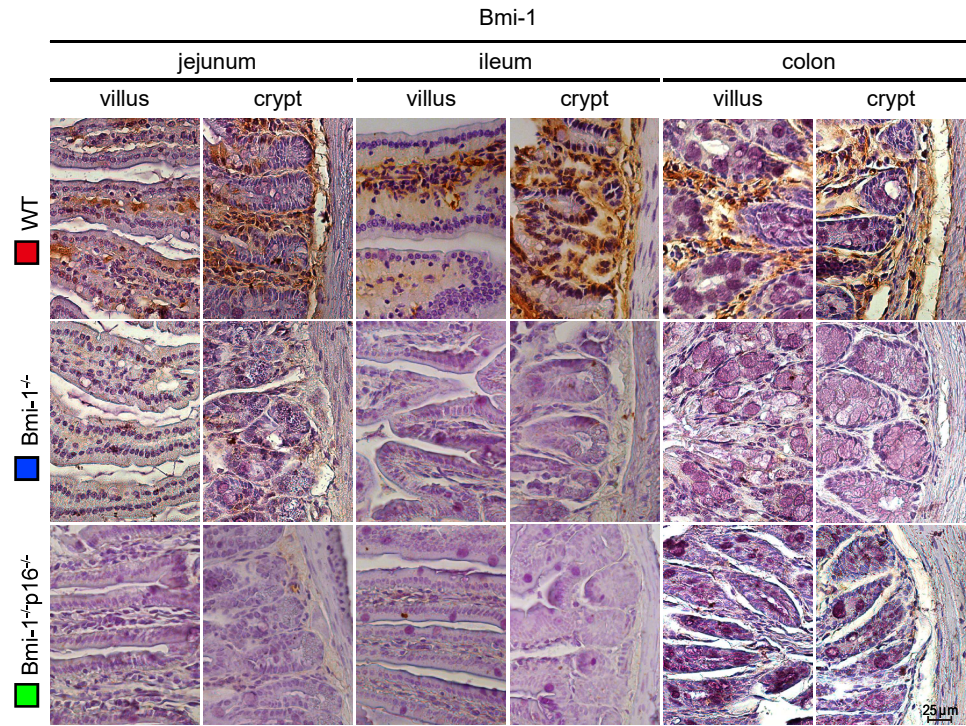

B.

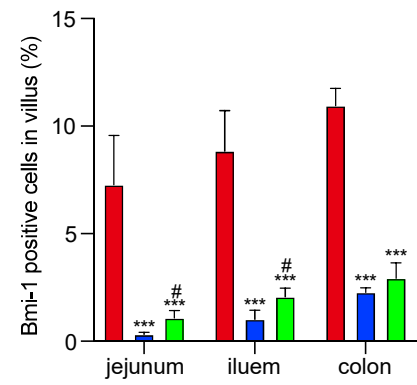

C.

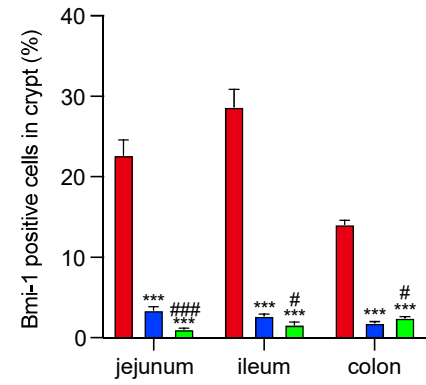

Figure S2

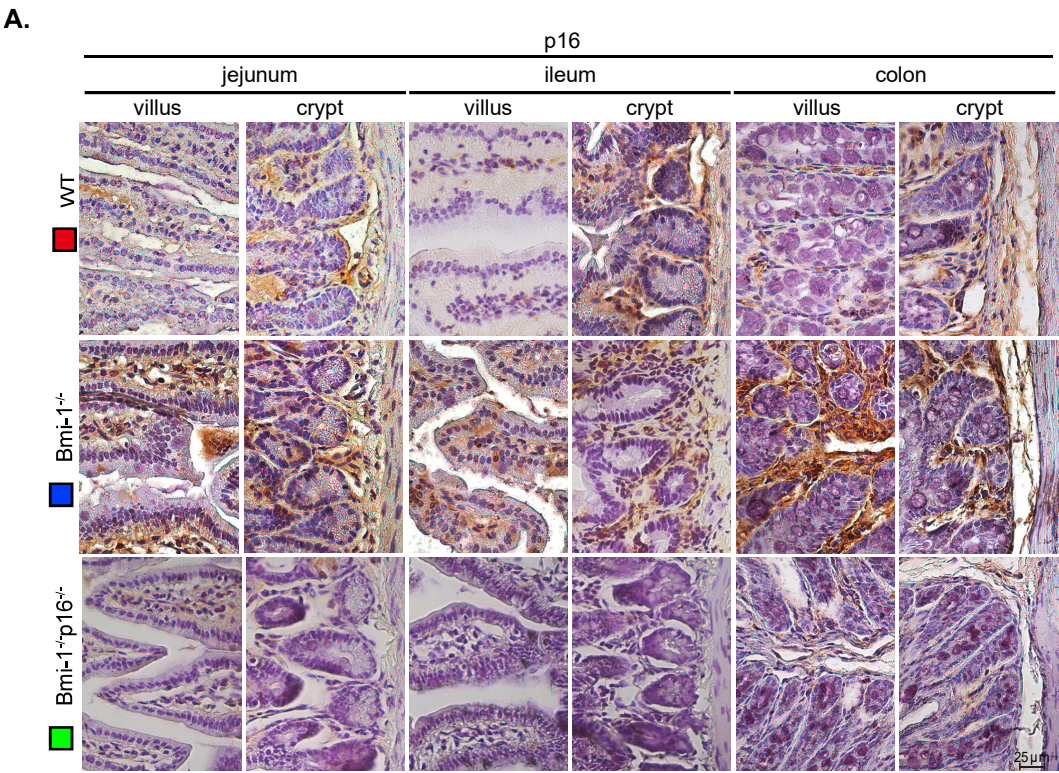

**B.**

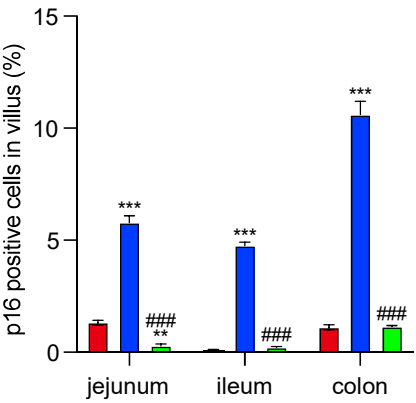

**C.**

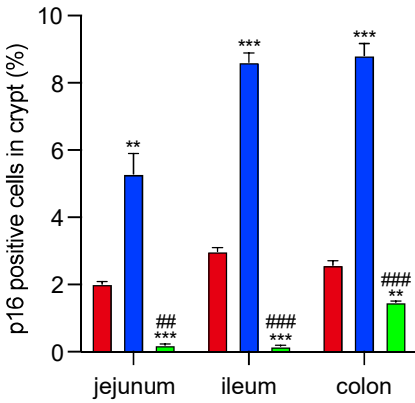

**Figure S3**

**A.**

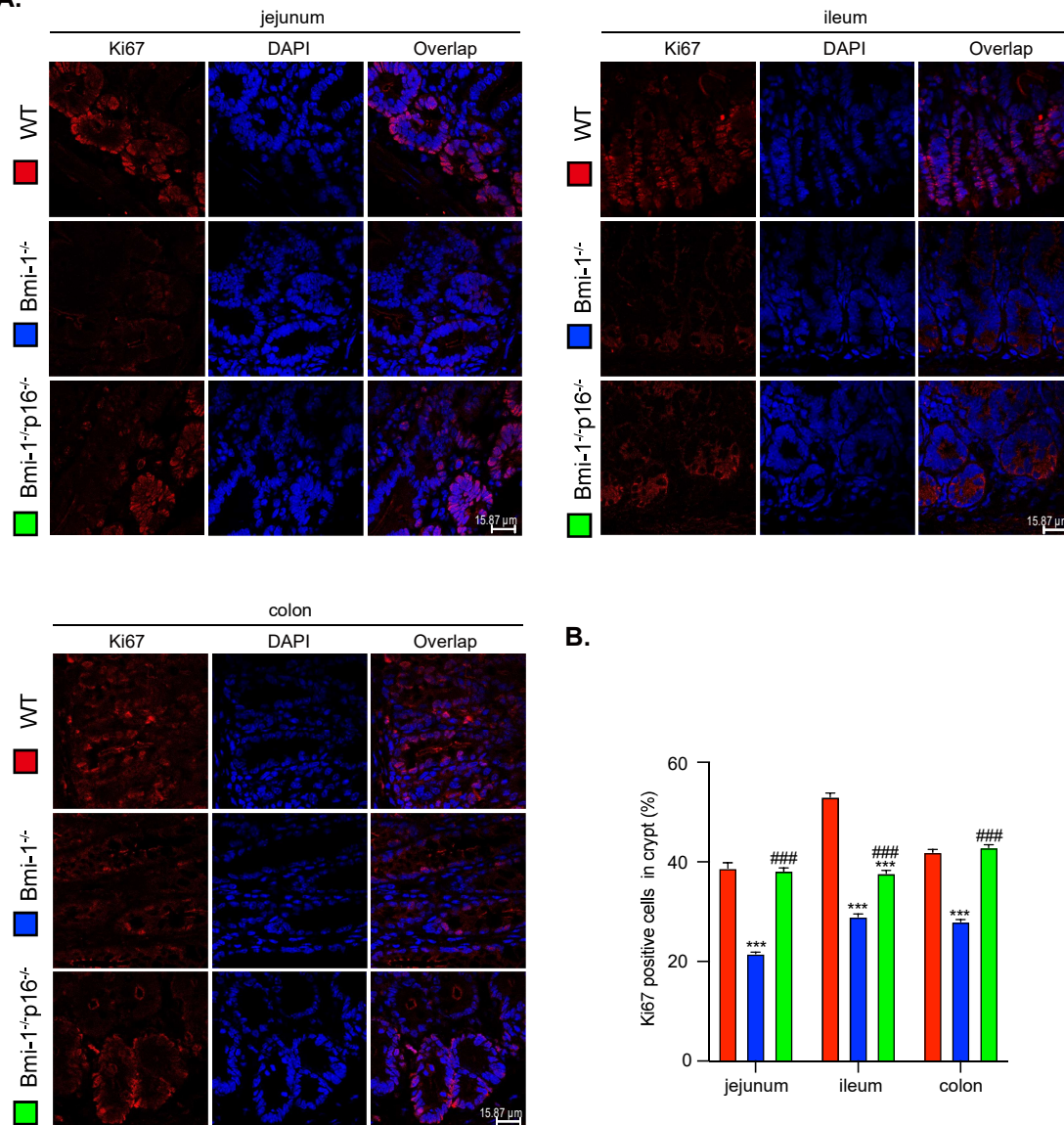

**B.**

Figure S4

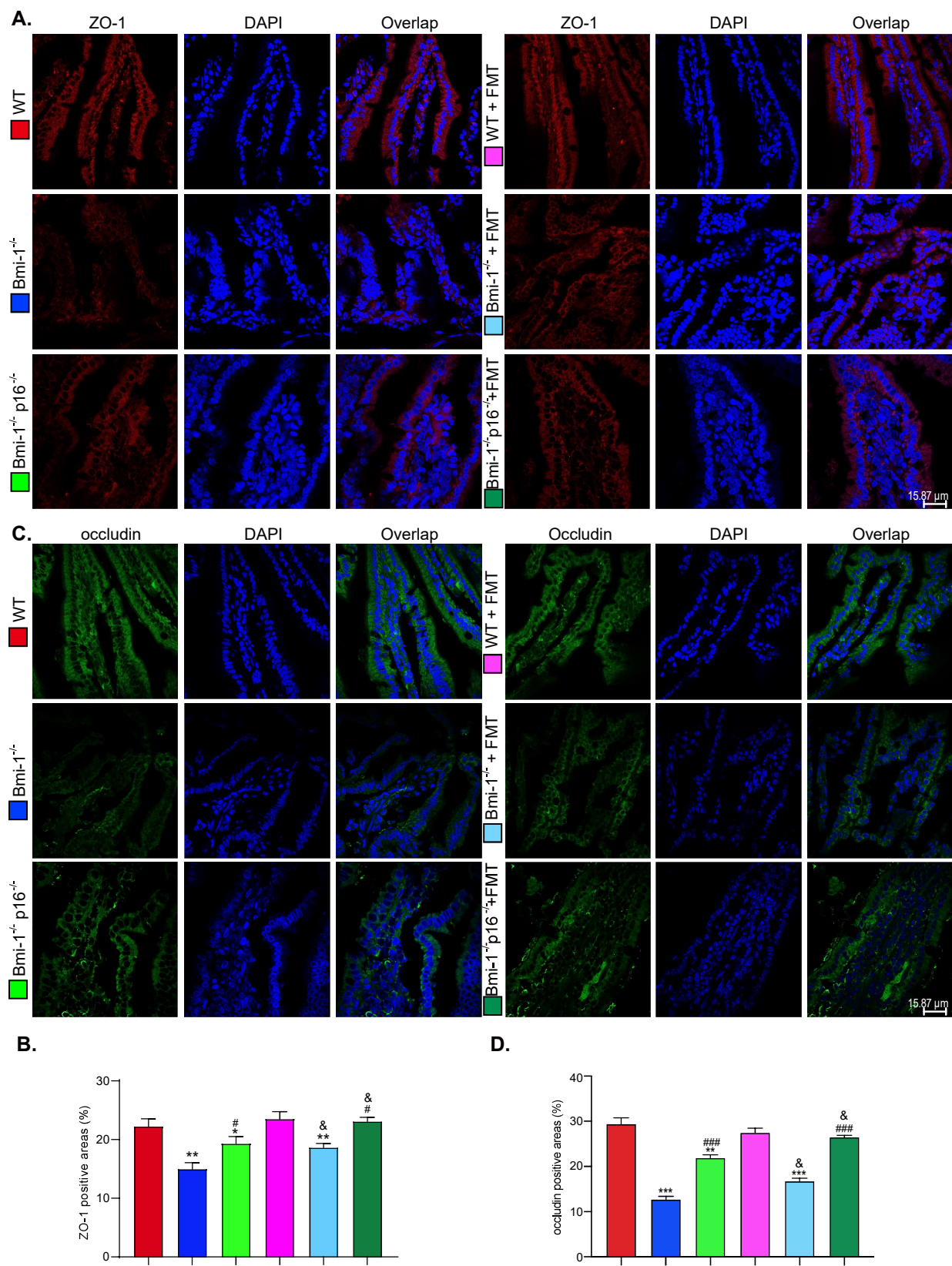

Supplement: Supplementary file 1 [file Data_Sheet_1.pdf]
