# Supplementary material for: P16INK4a Deletion Ameliorates Damage of Intestinal Epithelial Barrier and Microbial Dysbiosis in a Stress-Induced Premature Senescence Model of Bmi-1 Deficiency
Source: Front Cell Dev Biol. 2021 Oct 7;9:671564. doi: 10.3389/fcell.2021.671564 (PMC8545785; doi:10.3389/fcell.2021.671564)
Supplement: Supplementary file 2 [file Data_Sheet_2.docx]

**SI2 Figures S1-S4 Legends**

**Figure S1 Bmi-1 was widely expressed in epithelial cells of jejunum, ileum and colon**

The experiments were carried out on the 7-week-old *Bmi-1^-/-^*, *Bmi-1^-/-^p16^-/-^* and WT mice. (A) Representative micrographs of paraffin-embedded intestinal sections immunohistochemical staining for Bmi-1, with hematoxylin staining the nucleus. (B-C) Percentage of Bmi-1 positive cells relative to the total cells. Six mice per group were used for experiments. Statistical analysis was performed with One-way ANOVA test. Values are mean ± SEM from six determinations per group, ***P <0.001 compared with WT group; ^#^P <0.05, ^###^P <0.001 compared with *Bmi-1*^-/-^ group.

**Figure S2 P16 was widely expressed in epithelial cells of jejunum, ileum and colon**

The experiments were carried out on the 7-week-old *Bmi-1^-/-^*, *Bmi-1^-/-^p16^-/-^* and WT mice. (A) Representative micrographs of paraffin-embedded intestinal sections immunohistochemical staining for p16, with hematoxylin staining the nucleus. (B-C) Percentage of p16 positive cells relative to the total cells. Six mice per group were used for experiments. Statistical analysis was performed with One-way ANOVA test. Values are mean ± SEM from six determinations per group, **P <0.01, ***P <0.001 compared with WT group; ^##^P <0.01, ^###^P <0.001 compared with *Bmi-1*^-/-^ group.

**Figure S3 *P16* deletion improved the proliferation of intestinal cells in *Bmi-1^-/-^* mice**

The experiments were carried out on the 7-week-old *Bmi-1^-/-^*, *Bmi-1^-/-^p16^-/-^* and WT mice. (A) Representative micrographs showing immunofluorescent staining for Ki67 in the crypt of jejunum, ileum and colon, with DAPI staining the nucleus. (B) Percentage of Ki67 positive cells in crypt relative to total cells. Six mice per group were used for experiments. Statistical analysis was performed with One-way ANOVA test. Values are mean ± SEM from six determinations per group, ***P <0.001 compared with WT group; ^###^P <0.001 compared with *Bmi-1*^-/-^ group.

**Figure S4 Fecal microbiota transplantation (FMT) from WT mice ameliorated tight junction in intestinal epithelium of *Bmi-1*^-/-^ and *Bmi-1*^-/-^*p16*^-/-^ mice**

Fecal microbiota (FM) from three- to four-week-old WT mice (fecal samples 100 μl, 100 mg/ml) were transplanted to WT, *Bmi-1*^-/-^ and *Bmi-1*^-/-^*p16*^-/-^ mice by gavage every other day and lasted for 21 days. ZO-1 and occludin were determined by immunofluorescence staining. (A) Representative micrographs showing immunofluorescence for ZO-1 in intestinal epithelium of ileum, with DAPI staining the nuclei. (B) Percentage of ZO-1-positive areas (%). (C) Representative micrographs showing immunofluorescence for occludin in intestinal epithelium of ileum, with DAPI staining the nuclei. (D) Percentage of occludin-positive areas (%). Statistical analysis was performed with One-way ANOVA test. Values are mean ± SEM from six determinations per group, *P <0.05, **P <0.01, ***P <0.001 compared with WT or WT+FMT group; ^#^P <0.05, ^###^P <0.001 compared with *Bmi-1*^-/-^ or *Bmi-1*^-/-^+FMT group, ^&^P <0.05 compared with the same genotype mice without FMT.
