# Supplementary material for: P16INK4a Deletion Ameliorates Damage of Intestinal Epithelial Barrier and Microbial Dysbiosis in a Stress-Induced Premature Senescence Model of Bmi-1 Deficiency
Source: Front Cell Dev Biol. 2021 Oct 7;9:671564. doi: 10.3389/fcell.2021.671564 (PMC8545785; doi:10.3389/fcell.2021.671564)
Supplement: Supplementary file 3 [file Data_Sheet_3.docx]

**SI3 Table S1 Primer for Real time RT-PCR**

| Name | S/AS | Sequence | Species | Tm  (℃) | Length  (bp) |
| --- | --- | --- | --- | --- | --- |
| *IL-1β*  *IL-6*  *TNF-α*  *p16*  *ELMO1*  *MCP-1*  *(Ccl2)*  *NLRP3*  *β-Actin* | S  AS  S  AS  S  AS  S  AS  S  AS  S  AS  S  AS  S  AS | 5’-CTGGTACATCAGCACCTCAC-3’  5’-AGAAACAGTCCAGCCCATAC-3’  5’-TGTATGAACAACGATGATGCACTT-3’  5’-ACTCTGGCTTTGTCTTTCTTGTTATCT-3’  5’-AGTGACAAGCCTGTAGCCC-3’  5’-GAGGTTGACTTTCTCCTGGTAT-3’  5’-CCCGATTCAGGTGATGATGAT-3’  5’-GCGGGAGAAGGTAGTGG-3’  5’-GAGAACAGCAGCCGAGAAGAT-3’  5’-GTTGCAGGTCTCACTAGGCAG-3’  5’-TTAAAAACCTGGATCGGAACCAA-3’  5’-GCATTAGCTTCAGATTTACGGGT-3’  5’-GACCAGGTTCAGTGTGTTTT-3’  5’-GGTTGGTGCTTAGACTTGAG-3’  5’-GGCTGTATTCCCCTCCATCG-3’  5’-CCAGTTGGTAACAATGCCATGT-3’ | mouse  mouse  mouse  mouse  mouse  mouse  mouse  mouse | 60  60  60  56  56  56  60  60 | 124  197  252  100  116  120  130  154 |

S, sense; AS, antisense, sequence; Tm, annealing temperature; length, amplicon
