# Supplementary material for: P16INK4a Deletion Ameliorates Damage of Intestinal Epithelial Barrier and Microbial Dysbiosis in a Stress-Induced Premature Senescence Model of Bmi-1 Deficiency
Source: Front Cell Dev Biol. 2021 Oct 7;9:671564. doi: 10.3389/fcell.2021.671564 (PMC8545785; doi:10.3389/fcell.2021.671564)
Supplement: Supplementary file 4 [file Data_Sheet_4.docx]

**SI4 Alignment of occludin, MAPK8 and MAPK10 in human**


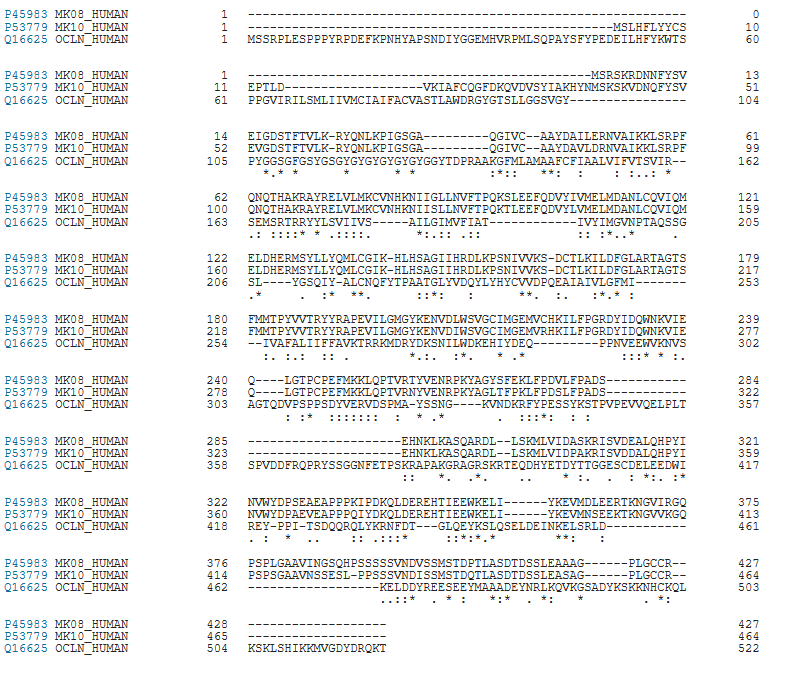


“*” indicating a single and fully conserved residue, “:” indicating residue with very similar properties, “.” indicating residue that is weakly similar.
