# Supplementary material for: P16INK4a Deletion Ameliorates Damage of Intestinal Epithelial Barrier and Microbial Dysbiosis in a Stress-Induced Premature Senescence Model of Bmi-1 Deficiency
Source: Front Cell Dev Biol. 2021 Oct 7;9:671564. doi: 10.3389/fcell.2021.671564 (PMC8545785; doi:10.3389/fcell.2021.671564)

**SI6 Reports of Occludin Plasmids**

1. Occludin-full length


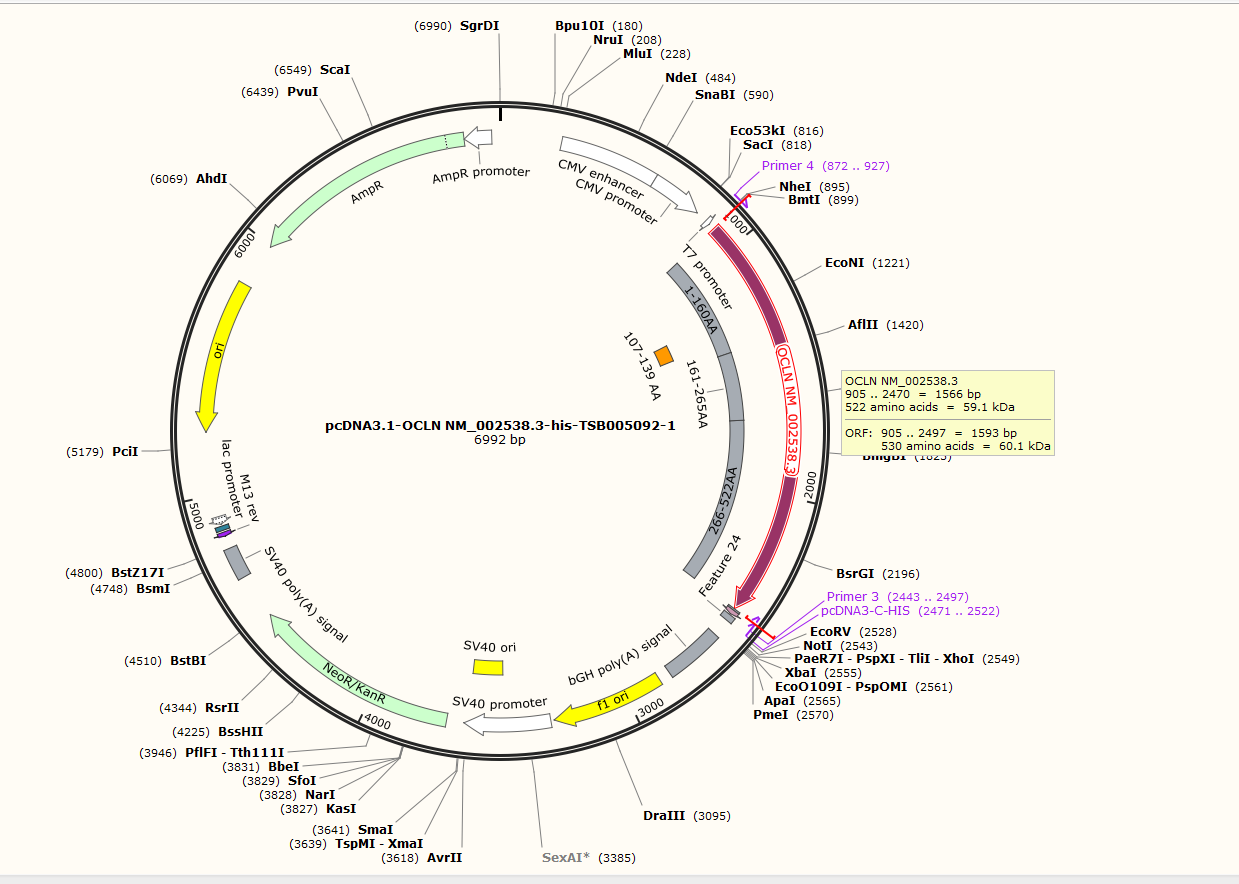


2. Occludin △1-160


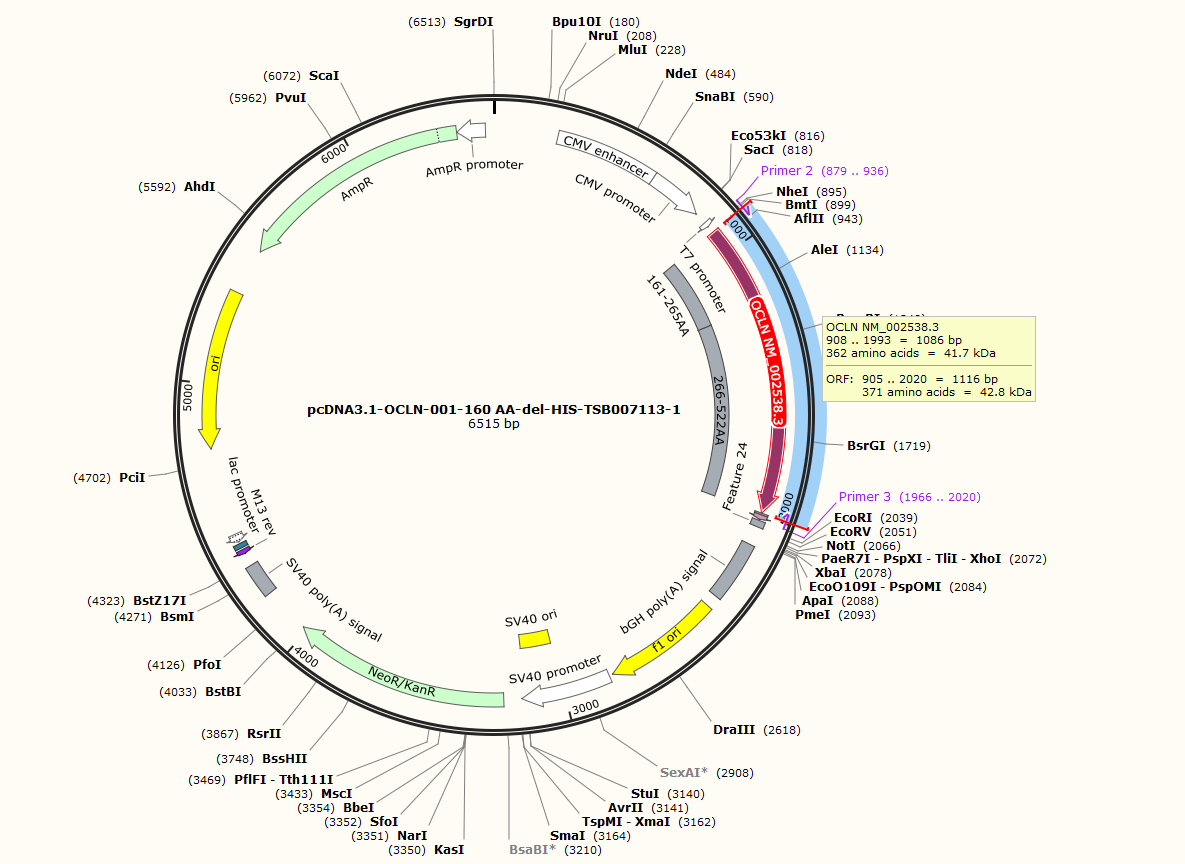


3. Occludin △161-265


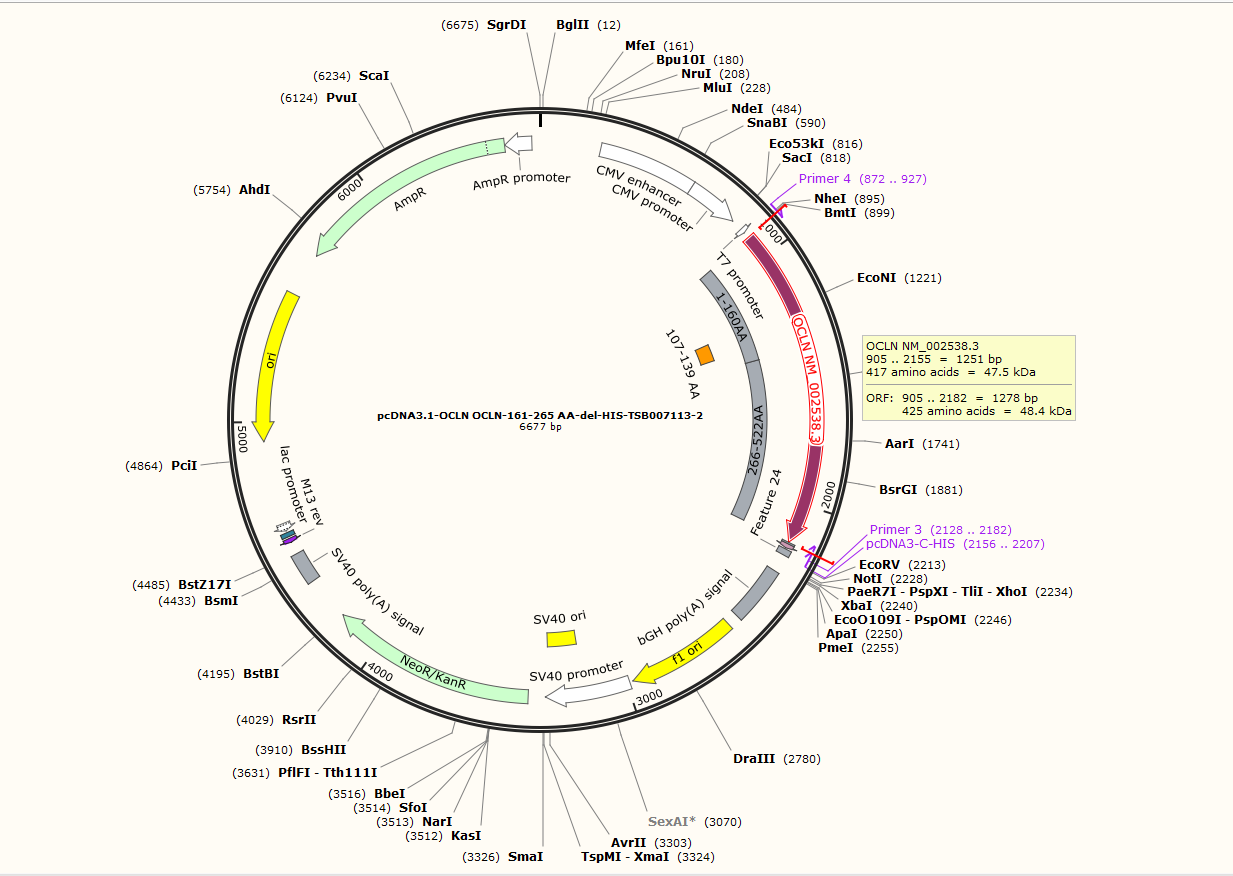


4. Occludin △266-522


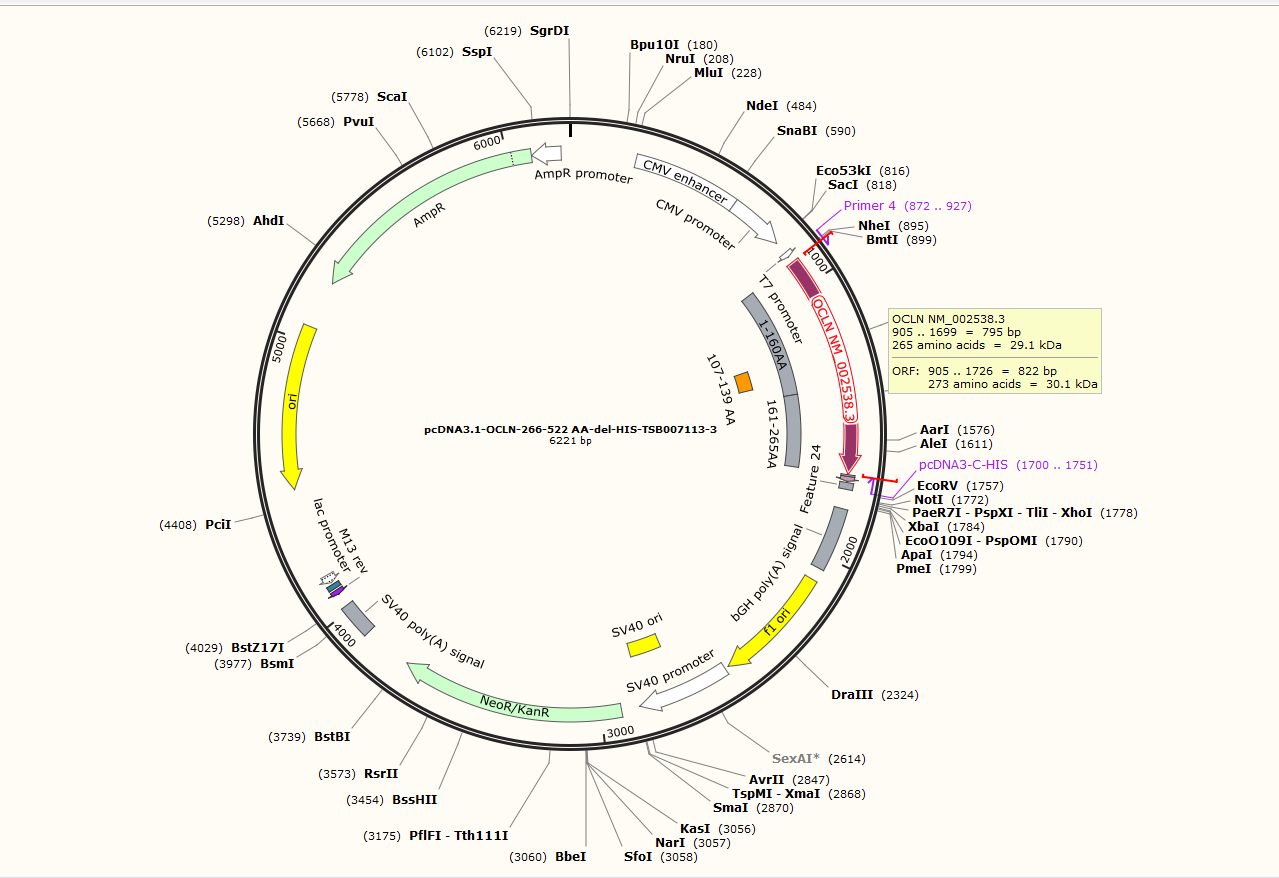


5. Occludin △107-139


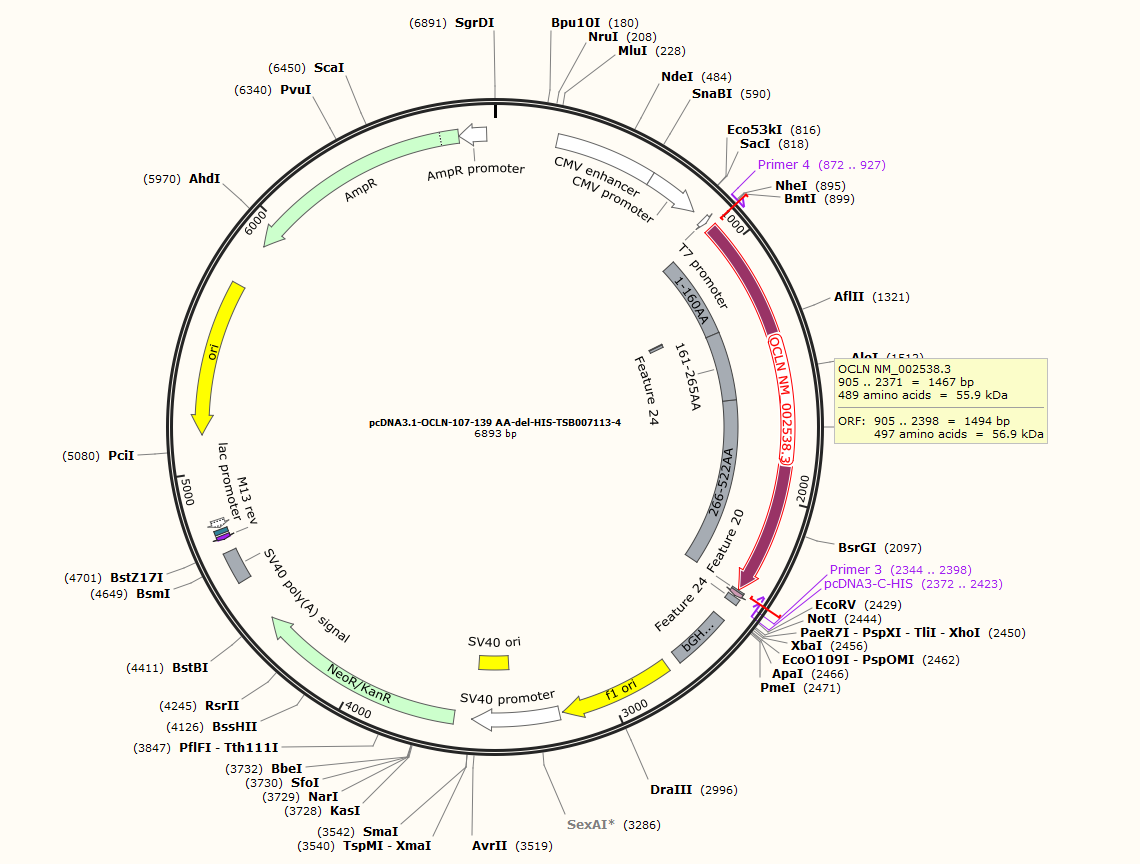


1. Occludin 266-522


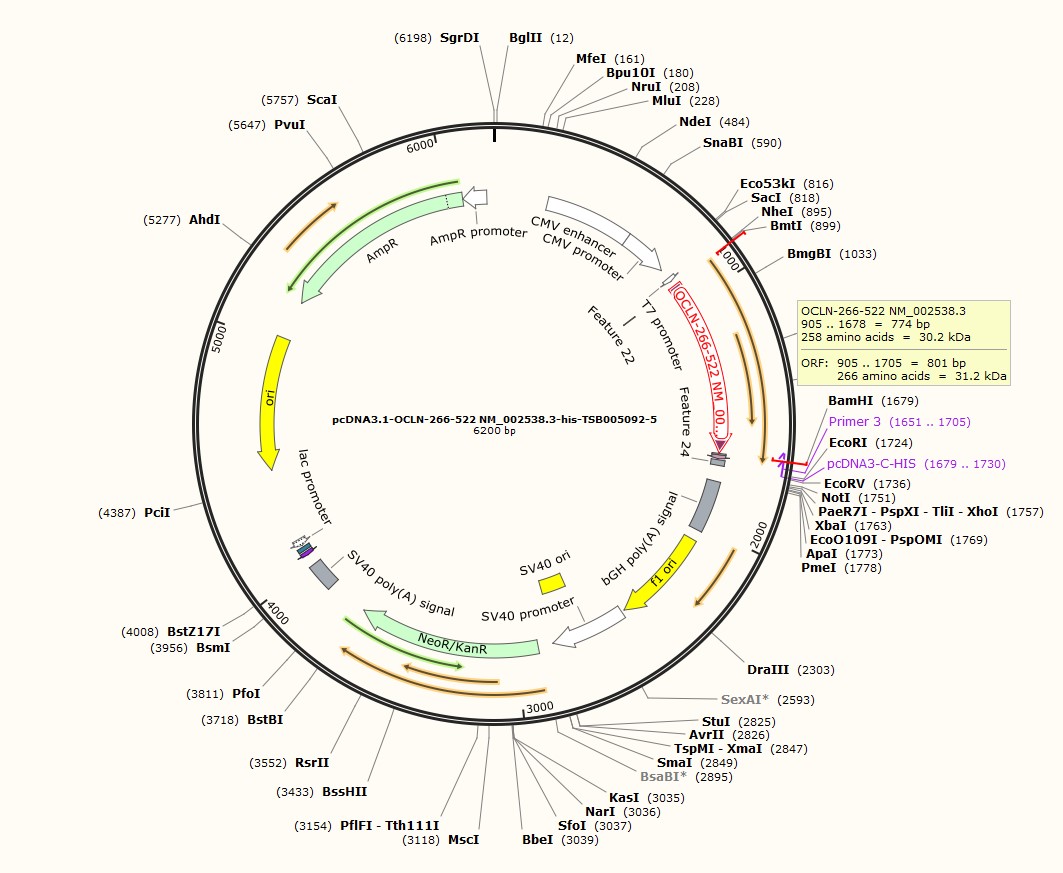


1. Molecular weight of occludin (Del 161-265) calculated the molecular weight using *Peptide and Protein Molecular Weight Calculator*

(https://www.aatbio.com/tools/calculate-peptide-and-protein-molecular-weight-mw)


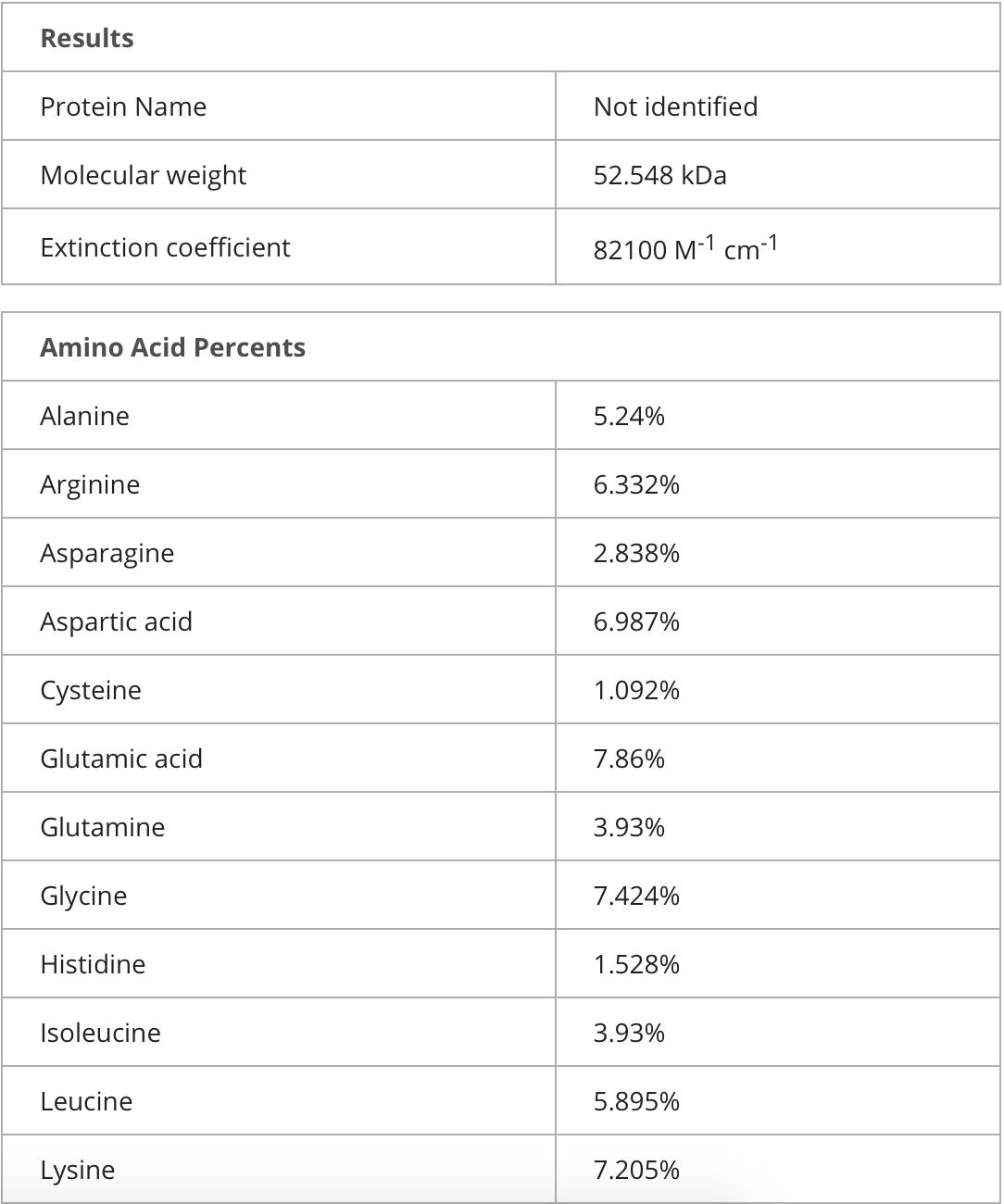


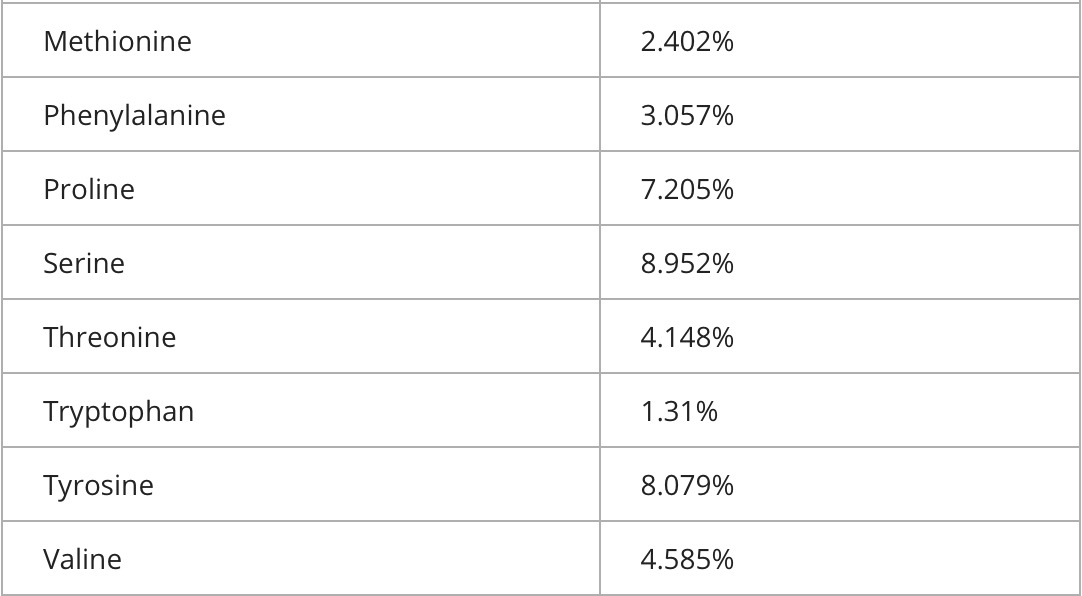

Supplement: Supplementary file 6 [file Data_Sheet_6.docx]
